# AbAMPdb: a database of *Acinetobacter baumannii* specific antimicrobial peptides

**DOI:** 10.1093/database/baae096

**Published:** 2024-10-12

**Authors:** Farha Anwer, Ahmad Navid, Fiza Faiz, Uzair Haider, Samavi Nasir, Muhammad Farooq, Maryam Zahra, Anosh Bano, Hafiza Hira Bashir, Madiha Ahmad, Syeda Aleena Abbas, Shah E Room, Muhammad Tariq Saeed, Amjad Ali

**Affiliations:** Atta-ur-Rahman School of Applied Biosciences (ASAB), National University of Sciences and Technology (NUST), Sector H-12, Islamabad 44000, Pakistan; School of Interdisciplinary Engineering & Sciences (SINES), National University of Sciences and Technology (NUST), Sector H-12, Islamabad 44000, Pakistan; Atta-ur-Rahman School of Applied Biosciences (ASAB), National University of Sciences and Technology (NUST), Sector H-12, Islamabad 44000, Pakistan; Atta-ur-Rahman School of Applied Biosciences (ASAB), National University of Sciences and Technology (NUST), Sector H-12, Islamabad 44000, Pakistan; Atta-ur-Rahman School of Applied Biosciences (ASAB), National University of Sciences and Technology (NUST), Sector H-12, Islamabad 44000, Pakistan; Department of Medical Lab Technology, BIC, University of Harīpur, Haripur, Khyber Pakhtunkhwa 22620, Pakistan; Atta-ur-Rahman School of Applied Biosciences (ASAB), National University of Sciences and Technology (NUST), Sector H-12, Islamabad 44000, Pakistan; Atta-ur-Rahman School of Applied Biosciences (ASAB), National University of Sciences and Technology (NUST), Sector H-12, Islamabad 44000, Pakistan; Atta-ur-Rahman School of Applied Biosciences (ASAB), National University of Sciences and Technology (NUST), Sector H-12, Islamabad 44000, Pakistan; Atta-ur-Rahman School of Applied Biosciences (ASAB), National University of Sciences and Technology (NUST), Sector H-12, Islamabad 44000, Pakistan; Atta-ur-Rahman School of Applied Biosciences (ASAB), National University of Sciences and Technology (NUST), Sector H-12, Islamabad 44000, Pakistan; Xylexa Inc, National University of Sciences and Technology (NUST), Sector H-12, Islamabad 44000, Pakistan; School of Interdisciplinary Engineering & Sciences (SINES), National University of Sciences and Technology (NUST), Sector H-12, Islamabad 44000, Pakistan; Atta-ur-Rahman School of Applied Biosciences (ASAB), National University of Sciences and Technology (NUST), Sector H-12, Islamabad 44000, Pakistan; MGBIO (SMC-PRIVATE) Limited, C4 H Building 1, National Science and Technology Park, NUST, H-12, Islamabad 44000, Pakistan

## Abstract

*Acinetobacter baumannii* has emerged as a prominent nosocomial pathogen, exhibiting a progressive rise in resistance to therapeutic interventions. This rise in resistance calls for alternative strategies. Here, we propose an alternative yet specialized resource on antimicrobial peptides (AMPs) against *A. baumannii*. Database ‘AbAMPdb’ is the manually curated collection of 300 entries containing the 250 experimental AMP sequences and 50 corresponding synthetic or mutated AMP sequences. The mutated sequences were modified with reported amino acid substitutions intended for decreasing the toxicity and increasing the antimicrobial potency. AbAMPdb also provides 3D models of all 300 AMPs, comprising 250 natural and 50 synthetic or mutated AMPs. Moreover, the database offers docked complexes comprising 5000 AMPs and their corresponding *A. baumannii* target proteins. These complexes, accessible in Protein Data Bank format, enable the 2D visualization of the interacting amino acid residues. We are confident that this comprehensive resource furnishes vital information concerning AMPs, encompassing their docking interactions with virulence factors and antibiotic resistance proteins of *A. baumannii*. To enhance clinical relevance, the characterized AMPs could undergo further investigation both in *vitro* and *in vivo*.

**Database URL**: https://abampdb.mgbio.tech/

## Introduction

Antibiotic resistance in microorganisms undermine ages of global efforts to combat infectious diseases [1]. *Acinetobacter baumannii* is listed as top priority pathogen by Centers for Disease Control and Prevention [2]. A Gram-positive bacterium that causes infections corresponds to the high rates of morbidity and mortality worldwide [3, 4]. *Acinetobacter baumannii* is more common in healthcare settings and a major cause of respiratory infections, urinary tract infections, ventilator associated pneumonia, septicaemia, endocarditis, wound infections, meningitis, and bloodstream infections [5]. Therefore, there is an urgent need to control this fast-growing antimicrobial-resistant *A. baumannii*via effective antimicrobials as it is showing resistance to available drugs such as vancomycin and aminoglycosides [6]. This diversion has also led the researchers to search for the alternative drugs and other therapeutic options, such as antimicrobial peptides (AMPs) to control this resistant pathogen.

According to modern therapeutics, AMPs have been regarded as a potent means of combating the antimicrobial resistance [7]. AMPs are a broad group of short cationic peptides and are produced by a range of organisms, including bacteria, archaea, protists, fungi, mammals, and plants with antibacterial and immunomodulatory properties, as well as wound healing and apoptosis induction [8]. The natural AMP melittin was first discovered back in 1952, by Habermann *et al*. from bee venom, and these natural AMPs are considered to be important parts in the defence system of their hosts [9].

Generally, AMPs have shown promising activity against Gram-positive and Gram-negative bacteria with less chances of resistance emergence as compared to antibiotics [7]. Despite the potential benefits of the natural AMPs, their effectiveness in clinical settings still facing challenges and mostly failed in clinical trials [10]. However, the emergence of specifically targeted AMPs [11], synthetic or mutated peptides [12], heralds a new era in antimicrobial treatment.

The research on AMPs to comprehensively optimize and explore their potential has led to the discovery of over 4000 natural AMPs. These reported AMPs have been catalogued in a variety of AMPs databases (both broad or narrow focused) including the Antimicrobial Peptide Database 3 (APD3) [13], Database of Antimicrobial Activity and Structure of Peptides (DBAASP) [14], ADAPTABLE [15], DRAMP 3 [16], and Collection of Anti-Microbial Peptides (CAMP) R4 [17]. While some narrow-focused databases have also been published such as defensins knowledgebase, which focusses specifically on collecting defensins-based peptides [18], Antiviral Peptides Database (AVPdb) for antiviral peptides [19], ParaPep designed specifically for antiparasitic peptides [20], BACTIBASE designed to characterize only bacteriocins [21], and LABiocin for lactic acid bacteria [22]. We noticed a gap in above databases when it comes to bacterial pathogen-specific AMP databases.

Therefore, a specialized resource, AbAMPdb, created especially for AMPs against *A. baumannii*, can help one explore the structural and functional connections as well as different applications of the AMPs. AbAMPdb is a manually curated database developed as a valuable resource for *A. baumannii* therapeutics, presenting the pre-screened and selected *A. baumannii*-specific natural AMPs derived from various resources (databases and literature) and the modified form of those natural peptides. These modified AMPs are created through a series of mutations or substitutions of specific amino acids—for instance replacing asparagine (N), tryptophan (W), valine (V), leucine (L), methionine (M), phenylalanine (F), histidine (H), tyrosine (Y), glutamic acid (E), and aspartic acid (D) with lysine (K), proline (P), cysteine (C), and isoleucine (I), respectively—using the amino acid walking method in a 5′ to 3′ orientation [12].

Overall, the AbAMPdb provides the AMP sequences, physiochemical properties associated with biological activities of the corresponding AMPs and their molecular structures, as well as the docked complexes between AMPs and the target virulence and antibiotic resistance associated proteins of *A. baumannii*. We believe that AbAMPdb will accelerate the research and design of novel AMPs, which serve as an effective anti-*A. baumannii* therapeutics option to further aid in clinical outcomes.

## Material and methods

### Data collection and organization

To stipulate complete and in-depth information on AMPs, we manually curated data from broad range of databases (primary and AMPs accessible databases), literature. To meet the evaluation criteria of AbAMPdb, AMPs with the following characteristics were included in the dataset: (i) less than 50 amino acid length, since it has been reported to be more stable; (ii) *A. baumannii* specific AMPs; (iii) activity of AMPs; (iv) data types (Experimentally validated/predicted); and (v) minimum inhibitory concentration. Total 350 AMPs were collected against *A. baumannii* from different resources. We extracted AMPs majorly using different keyword search terms like ‘antimicrobial peptides’, ‘antibacterial peptides’, ‘antibacterial peptides against *A. baumannii*’, ‘antimicrobial peptides against *A. baumannii*’ from different databases.

The major resources from which we extracted AMPs by using different keyword like, ‘Antimicrobial peptide’, ‘antibacterial peptide’, ‘antibacterial peptides against *A. baumannii*’, ‘antimicrobial peptides against *A. baumannii*’ from different origins and variety of sources including journals, AMP databases, such as DRAMP [16] [http://dramp.cpu-bioinfor.org/ (accessed on 1 October 2021)], DBAASP [14] [https://dbaasp.org/ (accessed on 1 October 2021)], CAMP [23] [http://www.camp.bicnirrh.res.in/ (accessed on 1 October 2021)], LAMP [24] [http://biotechlab.fudan.edu.cn/database/lamp/ (accessed on 1 October 2021)], APD 3 [13] [https://aps.unmc.edu/ (accessed on 1 October 2021)], and BaAMPs [http://www.baamps.it/ (accessed on 1 October 2021)] [25]. The collected AMPs provided in Supplementary Table S1 were then subjected to the physicochemical and biological parameter analysis.

### Physicochemical and biological parameter analysis

Peptide information collected for *A. baumannii*-specific peptides including sequence, length, MIC, and AMP type (Experimental/predicted). To analyse antimicrobial activity of AMPs, physicochemical parameters were selected based on AMPs gathered from different sources. A number of properties affect AMPs antimicrobial activity: amino acid composition, molecular weight (<10 KDa), length, net charge (>+2), and hydrophobicity for ascertaining the penetration ability of a peptide, and assuming the penetration ability of peptides and hydrophobicity, the isoelectric point was 10, instability index, aliphatic index, Boman index has the lowest value, representing the ability of a peptide to bind to another protein, which in turn influences the antimicrobial activity, amphipathicity, hydrophobic moment, haemolytic activity, antimicrobial activity, toxicity, and half-life of AMPs (in intestine-like conditions). Total 14 physicochemical parameters were analysed using the R peptide package [26] (https://cran.r-project.org/web/packages/Peptides/index.html (accessed on 15 January 2023)] that is specifically designed for AMPs, protean 3D [27] [https://www.dnastar.com/software/lasergene/protean-3d/ (accessed on 20 January 2022)], ProtParam tool [28] [https://web.expasy.org/protparam/ (accessed on 22 January 2022)], Amplify [29] [https://github.com/bcgsc/AMPlify (accessed on 30 January 2022)] is a deep learning python based package used to predict the antimicrobial activity of AMP, HemoPI [30] [https://webs.iiitd.edu.in/raghava/hemopi/standalone.php (accessed on 5 February 2022)] is a java based standalone tool to predict haemolytic activity of peptide without the limitation of number of amino acid residues and petide.bio [31] v0.19.0 [https://peptide.bio/ accessed on 5 November 2023) for haemolytic activity prediction of experimental AMPs due to their exceeded length and cannot be predicted by HemoPI and ToxinPred server [32] [http://crdd.osdd.net/raghava/toxinpred/ (accessed on 5 February 2022)]. The evaluated AMPs from physicochemical property assessment were integrated to AbAMPdb. Shortlisted peptides were used to construct synthetic library of AMPs.

### Construction and prediction of synthetic AMP

By altering the natural amino acid sequence of natural AMPs, the efficacy of natural AMPs can be enhanced. Five mutant peptides were created for each shortlisted AMP library (Experimental/natural AMP). As directed by Kumar *et al*., the strategy was adopted by substituting AMPs, which suggests following the 5ʹ → 3ʹ an amino acid walking method replacing amino acid residues such as asparagine (N), leucine (L), tryptophan (W), valine (V), methionine (M), phenylalanine (F), histidine (H), aspartic acid (D), tyrosine (Y), and glutamic acid (E) with lysine (K), proline (P), isoleucine (I), and cystine (C). For AMP design, the substitution of N, L, W, V, M, F, H, D, Y, and E was prioritized from numerous studies to demonstrate the minimum relevance. Maher and colleagues [33] suggested that by retaining its functional resemblances with the natural AMP, such substitutes are liable to increase antibacterial properties in AMPs in terms of overall charge, membrane permeability, and half-life. For further evaluation, five mutants for each peptide were selected based on the probability scores and subjected to the above-mentioned physicochemical and biological property analysis. The evaluated AMPs from physicochemical property assessment were integrated to AbAMPdb. The synthetic peptides along with the experimental peptides were further subjected to molecular docking analysis.

### 3D structure optimization for AMPs

Natural peptide and mutant/synthetic AMPs 3D structure were homology modelled by using AlphaFold v2.0 [34], an AI-based model that help in accurate prediction of protein structure. These structures were then integrated to AbAMPdb.

### Screening and optimization of antibiotic resistance genes and virulence proteins

Target proteins were selected based on their relevance to pathogenicity and resistance mechanisms, informed by literature and databases. We selected antibiotic resistance genes and virulence factors of *A. baumannii* from NCBI [35] [https://www.ncbi.nlm.nih.gov/ (accessed on 12 April 2022), PubMed [36] [https://pubmed.ncbi.nlm.nih.gov/ (accessed on 12 April 2022)], and UniProt [37] [https://www.uniprot.org/ (accessed on 13 April 2022)] databases. Total 20 candidates were selected based on their mechanism of antibiotic resistance and virulence, including OMP 33–36, OMPW, fimH, FBN, LptD, cat1, oprD, Oxa-51, adeA, adeC, adeR, adeS, ampC, baeR, baeS, bfmR, smpA, cmlA, strA, and sul1 provided in Supplementary Table S1.

The sequences were retrieved and subjected to homology modelling by trRosetta [https://yanglab.nankai.edu.cn/trRosetta/ [38] (accessed on 15 April 2022)], an online portal that builds the protein structure by direct energy minimizations with a restrained Rosetta algorithm. This homology-based platform working was based on the inter-residue distance and orientation distributions, predicted by a deep neural network.

### AMP-target protein docking

The synthetic AMPs along with their experimental AMPs were reflected as ligands. The experimental AMPs were docked with each target protein by using LightDock (V0.9.4) (https://github.com/LightDock/LightDock/releases/tag/0.9.4) (accessed on 1 October 2023) [39] for peptide–protein flexible docking that is based on swarm intelligence optimization algorithm and takes 1-min for the docking and integrated to AbAMPdb. The top proteins were selected based on interaction score to dock with synthetic peptides using LightDock (https://github.com/LightDock/LightDock/releases/tag/0.9.4) and CABSDock standalone (V0.9.18) (https://bitbucket.org/lcbio/cabsdock/wiki/Home (accessed on 3 January 2023) [40], a python-based toolbox that flexibly performs docking for a number of AMPs with each target protein.

### Rebuilding and refinement of synthetic AMPs’ docked complexes

The output model from CABSDock and LightDock simulations was then used for rebuilding the protein structure to an all-atom representation using the ca2all package. The rebuild model was further subjected to refinement by using Rosetta FlexPepDock, a high-resolution minimization protocol (https://www.rosettacommons.org/downloads/academic/3.13) (accessed on 13 February 2023) [41]. We ranked structures based on FlexPepDock energy terms score ‘total_score’, interface score ‘I_sc’, and bb_RMSD ‘rmsBB’. An interaction at the receptor/peptide interface is represented by ‘I_sc’, which represents the sum of energies contributed by these interactions. To rank the peptides, we consider the I_sc value, which could differentiate between the best and the worst peptides.

### AMP–protein interaction prediction

Discovery Studio® Visualizer 2022 [42] (Accelrys Software Inc., San Diego, CA, USA) and PyMOL [43], an open-source software designed by Warren Lyford DeLano for 3D structure visualization and ligand bond interaction. The Ligplot+ is used for 2D image visualization [44].

### AbAMPdb web interface and application

**Figure 1.**
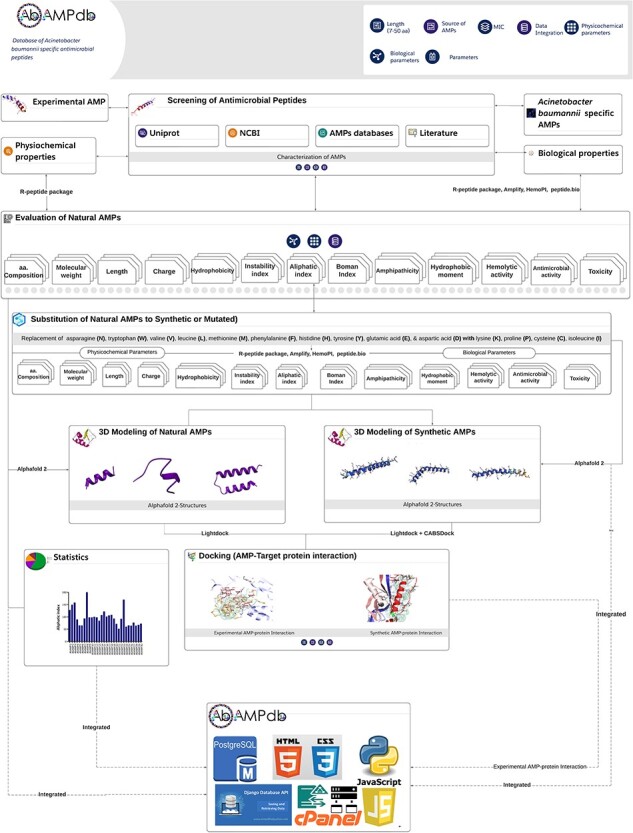
Methodology overview. The database development included four steps; screening of AMPS, evaluation of AMPs, homology modelling and target protein selection to dock with each AMP and integration of all the data into AbAMPdb.

The front-end of AbAMPdb was built using HTML, JQuery (V1.7.2), and Cascading Style Sheets and back-end used db.sqlite3 default Django. The web server and all parts of the database are hosted on cPanel shared hosting at the Integrative Biology and Genomics Group, Atta-ur-Rahman School of Applied Biosciences, National University of Sciences and Technology, Pakistan. The source code of AbAMPdb has been provided in GitHub as public repository (https://github.com/F-anwer/ampdb). The overall methodology is depicted in Fig. 1.

To facilitate the study of these peptides, two datasets have been created (experimentally validated and predicted), so users may restrict research to one or both datasets. To identify which sequence features contribute to the specificity and activity of AMPs, it is essential to focus on sequence, activity (MIC), and target organisms as well as non-target organisms. Literature references, taxonomy, and structural information can be used to gain a better understanding of AMPs. Therefore, AbAMPdb provides complete sequence and structure information.

## Results and discussion

### Database description

AbAMPdb comprises both natural (250) and synthetic or mutated (50) AMP sequences, which contain fewer than 60 amino acid residues.

The database provides basic information related to AMPs origin, sequence, physiological and biological properties, modelled 3D structures, and docked complexes of the AMPs and their target proteins. The desired properties of AMPs correspond to their effectiveness are calculated which comprise the specific amino acid composition, length, charge, hydrophobicity, haemolytic activity, isoelectric point, aliphatic index, instability index, Boman index, hydrophobic moment, and toxicity. The database can be accessed through URL: https://abampdb.mgbio.tech.

### Database interfaces and utility

**Figure 2.**
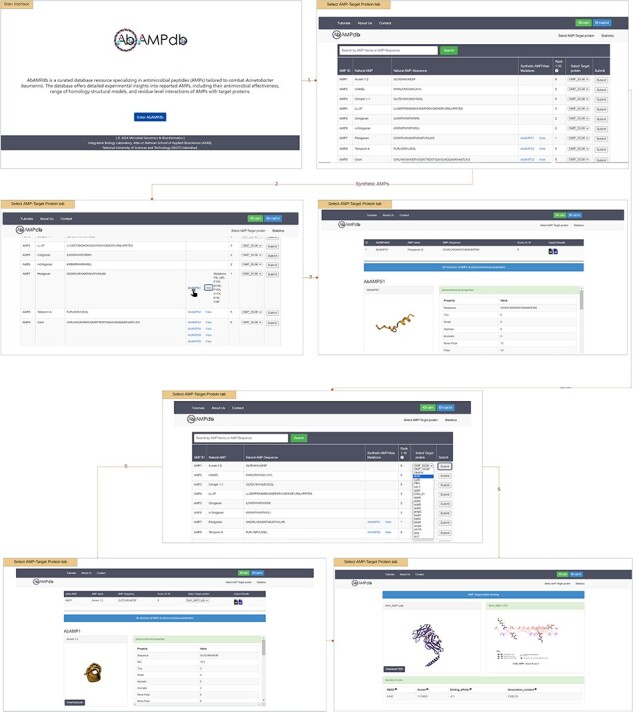
Web interface displaying the AbAMPdb.

**Figure 3.**
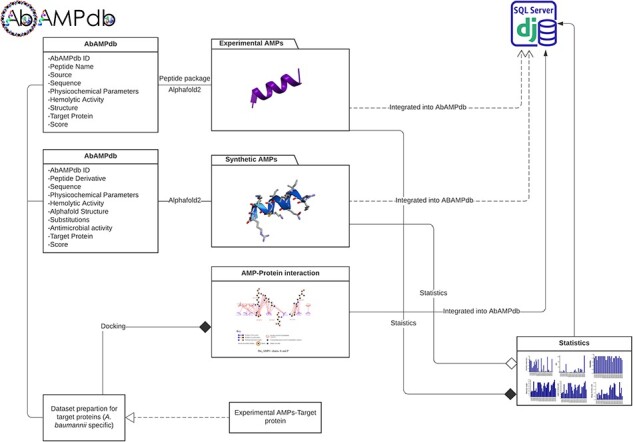
Architecture of AbAMPdb. The main architecture of the database depicting all types of data integration in the database.

The interface of AbAMPdb is carefully crafted to streamline AMPs-focused research and to meet clinical demands in combating the impact of *A. baumannii* on public health. It serves as a comprehensive platform, consolidating a compilation of AMPs sourced from literature and databases for easy access and reference on a single platform (Fig. 2). Architecture of database include Main interface, ‘Select AMP-Target protein’ tab, and ‘Statistics’ tab (Fig. 3) and search function is also included to search the AMP with a sequence or name to make the database user-friendly.

#### Select AMP-target protein

The provided feature of AbAMPdb offers users a comprehensive platform to access and analyse AMPs and their interactions with target proteins. User can view all 250 Natural AMPs by clicking ‘Select AMP-Target Protein tab’. The Synthetic AMPs can be viewed in ‘Synthetic AMPs/View Mutation’ column together with the natural AMPs on ‘Select AMP-Target Protein’ tab along with all the suggested mutations by clicking view option. The 3D structure and physicochemical properties of the selected AMP can be accessed by clicking the ID of synthetic AMP (displayed by hyperlink). Through a user-friendly interface, users can select target protein from a dropdown menu against the desired natural AMP. Upon clicking submit button user can view and download the 3D structure of AMP, and additionally can access the physicochemical properties. While scrolling down, users can download the PDB structure of docked complex, CSV and XML files containing physicochemical properties, root mean square deviation (RMSD), scores, binding affinity, and dissociation constant. Users can visualize the 2D interaction displayed on the right panel, this visualization of residue-level interactions enhances the understanding of AMP–protein binding mechanisms.

By correlating structural features with quantitative measurements, such as binding affinity and dissociation constants, researchers can gain deeper insights into the dynamics of AMP–protein interactions.

### Statistical description and findings

This feature includes the statistical information regarding AMPs based on their physicochemical parameters.

#### Physicochemical property identification

Physicochemical property analysis of AMPs unveils comprehensive understanding of physicochemical and biological features. Thorough analysis of natural and synthetic/mutated AMPs elucidates structural characteristics and antimicrobial efficacy of AMPs. Our results indicate that all of the integrated natural AMPs ranged between 8 and 60 amino acid (aa) residue in length, while synthetic or mutated AMPs ranged from 12 to 53 aa in length demonstrating rapid and potent antimicrobial effects compared to longer peptides [45].

It was found that 96% of the natural AMPs had a positive net charge with the average value of 5.46 while all the synthetic or mutated AMPs carried positive net charge with an average estimated value of 11.1. Because most AMPs are cationic, with a net positive charge range from +3 to +11, they can preferentially connect to the negatively charged surface of bacteria via electrostatic binding [46]. Most of the natural peptides possess hydrophobic content in range of 15% to 76.92%, while synthetic or mutated AMPs contain 25% to 50%, which favours an amphipathic conformation upon interaction with membranes [47].

It was found that 89% of natural AMPs were non-haemolytic peptides while all the synthetic or mutated AMPs carried 98% non-haemolytic peptides that contributed towards the reduced potential for damaging red blood cells [48]. Analysis of physicochemical properties revealed that 98% of natural AMPs exhibited an isoelectric point close to 10, indicating a predominantly basic nature. Additionally, AMPs demonstrated higher aliphatic index values, suggesting a tendency towards hydrophobicity. The hydrophobic moment ranged between 0.13 and 1.15, indicating varying degrees of amphipathicity among AMPs. Furthermore, lower Boman index values, with an average estimate of 1.3, were observed, suggesting reduced propensity for self-aggregation.

Importantly, the majority of AMPs were found to exhibit non-toxic properties, while the isoelectric point of all the synthetic or mutated AMPs was close to 10, with higher aliphatic indexes, hydrophobic moment ranged between 0.15 and 1, lower Boman index values, and all are non-toxins that shows high interaction with the membranes, heat stable, high transmembrane and phospholipid membrane interaction, high binding potential, and less toxic. The combination of high binding potential, membrane interaction, and low toxicity profile highlights the promising therapeutic properties of these synthetic or mutated AMPs [49].

MIC values have a greater contribution to the activity of AMPs and the majority of the shortlisted natural AMPs have a MIC lower than >250 µg/ml, indicating the potential activity of these AMPs [50]. Additionally, the AbAMPdb schema incorporated homology modelled 3D structures of relative AMPs and docking complexes of AMP-target proteins in Protein Data Bank (PDB) format. This method aligns with previous research that emphasizes the importance of integrating structural data into databases for AMPs [51]. It also extensively records statistics of AMPs alongside their corresponding physicochemical attributes aligning to existing methods for thorough peptide analysis. This comprehensive data plays a crucial role in elucidating the evolutionary connections among AMPs and their effectiveness against specific pathogens.

#### Anti-A. baumannii AMPs identification and evaluation

As naturally occurring AMPs may possess some undesirable characteristics such as instability due to proteases, high haemolytic activity, and high toxicity, etc. [52]. To overcome these drawbacks, synthetic AMPs (SAMPs) can be designed [53]. Designing synthetic or mutated AMPs peptides from natural AMP’s sequences have two aims: (i) to extract the better part, which holds antimicrobial activity; (ii) to exclude the worse part from that of a particular sequence, which is toxicity and low resistance to proteolysis [54]. One of the main strategies applied for designing synthetic or mutated AMPs by amino acid substitutions of N, L, W, V, M, F, H, D, Y, and E, which have little relevance in the design of AMPs with K, P, I, and C, have been shown to enhance antimicrobial activity. The 205 mutants were designed by substituting isoleucine (I), cysteine (C), proline (P) and lysine (K) instead of glutamic acid (E), tryptophan (W), aspartic acid (D), tyrosine (Y), asparagine (N), methionine (M), phenylalanine (F), valine (V), and leucine (L) in natural peptide sequences [12]. We utilized AMPlify [29] to predict antimicrobial activity of synthetic or mutant AMPs, a probability score ≥5 was considered as AMPs, whereas those with probability score <5 was considered as non-AMPs. There were 133 mutant AMPs with probability scores of >0.7 that subjected to be analysed for physicochemical properties. A further analysis of mutants for toxicity was conducted, and 50 mutants showed non-toxic properties. A total of 300 AMPs were categorized for further analysis, including 250 experimental and 50 synthetic or mutated AMPs resulting from substitutions of residues highlighted in red in Supplementary Table S2.

#### Ranking best AMPs

To specifically evaluate the peptide based on its bioactive ability, PeptideRanker tool (http://distilldeep.ucd.ie/PeptideRanker/) [55] was used to rank peptides. Total 250 experimental AMPs were ranked, and their probability scores were ranged between 0.5 and 1, as standard set by PeptideRanker for bioactive peptides. A value of 1 indicates high bioactive peptide [56] and 70% of natural peptides and 90% of synthetic or mutated peptides were shown high bioactivity. The AMPs were then subjected to the 3D structure optimization.

#### 3D structure optimization of AMPs

**Figure 4.**
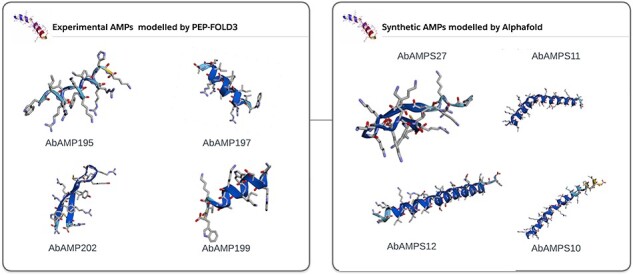
3D modelled structures of AMPs. 3D modelling of natural AMP and synthetic AMP.

In subsequent analysis, 70% of natural AMPs and 90% of synthetic or mutated AMPs showed high pLDDT as can be seen in Supplementary data. AlphaFold2 [34] accurately predicted the 3D structure (Fig. 4). pLDDT is a measure of confidence per residue which usually range between 0 and 100 and can be classified into four levels: a lowest pLDDT is 50, a low pLDDT is >70, a confident pLDDT is 70–90, and a high pLDDT is >90. As reported in various studies it has been shown that AlphaFold is of great importance in predicting protein structure as it gives resemblance of modelled structure with the crystal structure already present in the databases [57]. AlphaFold describes the atomic contacts between two atoms or ions using a string. We can simulate a protein molecule’s 3D atomic coordinates and compare them with its crystal structure using this concept. The AlphaFold’s predicted structure may significantly improve the docking confirmations due to peptide flexibility. The pLDDT information given by AlphaFold were included in the Supplementary Fig. S1. A total of 300 3D structures of AMPs were integrated into AbAMPdb and can be downloaded in PDB format. These 3D modelled AMPs were used for docking with target proteins (Fig. 4).

#### Binding energies from docking studies of AMPs

The selection of AMPs is based on a comprehensive analysis of physicochemical properties and docking interactions. The 3D modelled structure of 250 natural AMPs was used for docking via LightDock [39]. For AMPs screening, top 26 AMP candidates with favourable binding energy score ranges from −8 to −16.9 were selected. Among the screened AMPs, priority was given to AMPs capable of interacting diverse array of target proteins of *A. baumannii*.

Human KS-30 (Cathelicidin) and Temporin-PTa showed lowest values of binding affinities with fimH and sul1 having binding affinities −16.9 and −14.1 but the priority was given to AMPs that involves in interaction with diverse array of pathogenic proteins (virulence and antibiotic resistance). So, the five AMPs named as AMP20 (Cathelicidin-BF), AMP170 (NK-2), AMP172 (NK23a), AMP212 (CAME), and AMP234 (Alyteserin-2a) selected as top candidates based on PeptideRanker scores 0.7, 0.5, 0.7, 0.4, 0.6 and binding affinities in a range between −8 and −12.3. Top 5 interactions were selected and were included in Supplementary Table S3.

**Figure 5.**
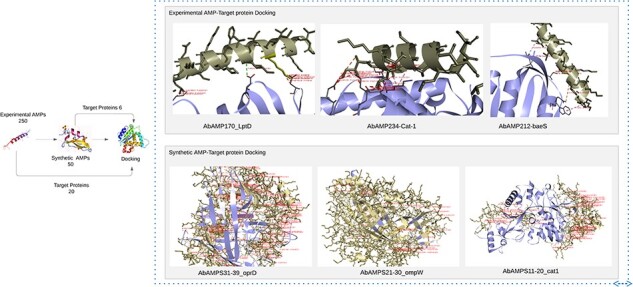
Docking of experimental AMP and synthetic AMP with pathogenic proteins of *A. baumannii* reveals top 6 AMPs that has low binding energies.

‘Cathelicidin-BF’ binds to OMPW, oprD, LptD, FBN, baeR, sul1, adeC; ‘NK-2’ exhibited affinity with strA, adeR, cat-1, oprD, adeA, LptD, OXA-51, FBN, fimH; ‘NK23a’ showed molecular interaction with smpA, adeS, oprD, baeS, LptD, OXA-51, FBN, adeC, ampC, fimH; ‘CAME’ displayed binding interaction with cmlA, omp33-36, cat-1, baeS, LptD, FBN; ‘Alyteserin-2a’ exhibited docking interaction with bfmR, adeS, cat-1, FBN, adeC, fimH having binding affinities in a range from −8 to −9.8, −8 to −11.5, −8 to −10, −8 to 10.5, −8.5 to 12.3, respectively. Subsequent analysis revealed that Top3 candidates namely NK-2, CAME, and Alyteserin-2a exhibited lowest values of binding affinity −11.5, −10.5, −12.3 with specific target proteins LptD, baeS, and cat-1, respectively indicating strong binding interactions, lowest binding affinity values, and high hydrophobic interactions, salt bridges, hydrogen bonds with the target proteins (Fig. 5) [58].

Due to resource constraints, CABSDock simulations was not applied to natural AMPs candidates as it took 3 days for one receptor with the entire set of 10 AMPs with single protein. To ensure timely analysis, we have selected synthetic or mutated AMPs docking simulation with six target proteins using CABSDock.

In parallel, we performed LightDock docking and simulations with the CABSDock for synthetic or mutated AMPs with six target proteins (cat1, oprD, adeR, ampC, omp33-36, ompW, and oxa51). CABSDock perform docking simulations at *n* = 1000 with multiple peptides at a time with single protein, so, we ran the docking simulation by making set of 10 synthetic or mutated peptides with each protein on cloud VM has 64gb core allotted to us.

Our results indicated that LightDock showed significant minimization of Interface score (I_sc) values following FlexPepDock refinement. The confirmations were ranked for productive binding modes based on the mean interface score ‘I_sc’ using FlexPepDock. Inspite of geometric constraints (binding modes), the interface score values show a considerable range. Top 3 combinations of synthetic or mutated AMPs give lowest I_sc value that suggests a strong binding interaction.

Docking simulations analysis was basically conducted to evaluate the target protein interaction with the synthetic/mutant AMPs. The CABSDock simulation results provide insights into the I_sc and RMSD before and after refinement for cat1 (Pexiganan-D-1, Temporin A-1, Human KS-30 (Cathelicidin)-3, KR-12 (Cathelicidin)-3), ompW (SAAP-148-1, AM-CATH36-4, AM-CATH21-2, Cathelicidin-BF-2, NA-CATH-1), and oprD (NA-CATH-2, WAM1-2, Bactenecin-1, HD5d5-2, Magainin-2-2).

**Figure 6.**
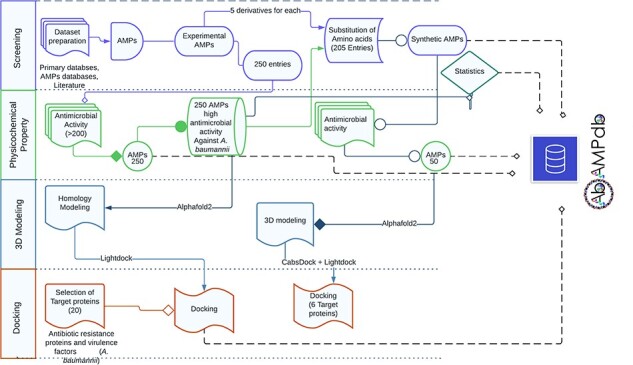
Overall statistics and findings.

Before refinement, the values of I_sc were 7768, 8465, 15 002 with a bb_RMSD of 0 while significant decrease was observed post-LightDock refinement, indicating improved binding interactions [59]. The bb_RMSD_after_Refinement shows stable RMSD values in range between 1 and 1.6 Å [60] highlighted in Supplementary Table S4. The comprehensive statistics elucidating overall statistics and finding of the methodology applied were elucidated (Fig. 6).

### Comparison between AbAMPdb and other databases

Over the last decade, more than 10 000 AMPs have been studied. Therefore, several databases were created to collect these AMPs and catalogue their associated information. Each AMP database stores specific information, tools, and statistics. Table 1 provides basics comparison between AbAMPdb and other AMP databases. As we witnessed, various databases have emerged over the past decade, presenting extensive collections of AMP sequences from diverse origins, including DAMPD [61], APD3 [13], and CAMP_R4_ [17]. Among them, APD3 is the most popular public collections of AMPs, harbouring 2625 mature sequences. Whereas DAMPD is a replacement of the ANTIMIC [62] database and has extended its entries to 1232, including both precursor and mature sequences. CAMP_R4_ holds 24 243 entries [experimentally validated (16 945) and predicted (7298)] and has integrated an antimicrobial activity prediction function based on machine learning algorithms. The number of databases having diverse origins played an important role in certain categories of AMPs; however, there are limitations on sequences collection. DBAASP v3 contains >15 700 AMPs sequences against broad targets [14], and YADAMP [63] is another database containing 2133 antibacterial and antifungal peptides. dbAMP 2 [64] holds 26 447 AMPs sequences that have antibacterial, antifungal or antiviral, antiparasitic activities. ADAPTABLE holds more than 40 000 entries and has targets like anticancer, antibacterial, antibiofilm, antiviral, antifungal, or antiparasitic [15]. DRAMP 3 contains 22 259 having antimicrobial, immunomodulatory, and other categories (anticancer, cytotoxicity, wound healing, etc.) of AMPs [16]. Additionally, there are more narrowly focused databases concentrating on specific types of peptides or organisms, such as the Defensins Knowledgebase encompasses records of 350 defensins that show activities against diverse organisms like fungi, viruses, Gram-positive and Gram-negative bacteria [18]. AVPdb covers 2683 peptides and provides detail information on antiviral peptides [19]. ParaPep holds 863 peptides against various species, including *Plasmodium, Leishmania*, and *Trypanosoma* [20], and LABiocin holds 517 bacteriocins mainly from Gram-positive and Gram-negative bacteria [22]. Furthermore, few of them focus on AMPs produced by plants, such as PhytAMP, which holds 271 plant based peptides with activities like, antibacterial, antiviral, or antifungal [65]; bacteria such as BACTIBASE holds 177 bacteriocins with main targets being Gram-positive and Gram-negative bacteria [21]; shrimp such as PenBase contains 200 entries of AMP’s sequences from diverse resources [66] cater to specific organisms or classes of organisms and LAMP that contains the cross-linking AMPs and currently holds 23 253 unique AMP sequences [24].

**Table 1.** Comparison of AbAMPdb with other available AMP databases

AMP Database	Search keywords	AMP origin	Data collection	Number of entries	Year	MIC data	Boman Index	Hydrophobic moment	Molecular docking	Organism/Class of organisms	References
AbAMPdb	Antibacterial, Antimicrobial, AMPs against *A. baumannii*	Natural	NCBI, UniProt, AMPs Databases	250 Natural + 50 Synthetic	2023	Present	Present	Present	Present	*A. baumannii*	
PENBASE	–	Shrimp	shrimp species	200	2008	Absent	Absent	Absent	Absent	Gram-positive bacteria and antifungal	1
BACTIBASE	Bacterial AMPs, eubacteria, peptide sequence	Bacteriocins produced by Gram-positive and Gram-negative bacteria	UniProt	177	2009	Present	Absent	Absent	Absent	Gram-positive and Gram-negative bacteria	2
ParaPep	Antimalarial peptides, antiparasitic peptides, antiplasmodial peptides etc	Natural	Literature, AMP’s databases	863	2014	Absent	Absent	Absent	Absent	*Plasmodium, Leishmania* and *Trypanosoma*	3
PHYTAMP	–	Plant	UniProt, Literature	271	2009	Present	Absent	Absent	Absent	Gram-positive and Gram-negative bacteria, fungi and viruses	4
DEFENSIN kNOWLEDGEBASE	Defensin	Defensins	UniProt, GenBank	350	2008	Absent	Absent	Absent	Absent	Gram-positive and Gram-negative bacteria, fungi and viruses	5
LABiocin	LAB bacteriocins, antibacterial peptide	Bacteriocins	Bactibase, BAGEL3	517	2019	Absent	Absent	Absent	Absent	Gram-positive and Gram-negative bacteria	6
DAMPD	Antifungal, Antiparasitic, Anticancer, Antimicrobial,	Natural	UniProt	1232	2011	Absent	Absent	Absent	Absent	Gram-positive and Gram-negative bacteria	7
APD3	Antiparasitic, Antifungal Antimicrobial, Anticancer,	Natural	GenBank, UniProt	1228	2009	Present	Present	Absent	Absent	Gram-positive and Gram-negative bacteria, fungi, viruses, parasites	8
YADAMP	–	Antibacterial	UniProtKB/Swiss-Prot, APD, CAMP	2133	2012	Present	Present	Present	Absent	Gram-positive and Gram-negative bacteria, and fungi	9
CAMPR4	Antimicrobial, Antifungal, Anticancer, Antiparasitic, antiviral	Natural & Synthetical	NCBI protein, UniProt, PubMed, Lens	24 243	2010	Present	Absent	Absent	Absent	Gram-positive and Gram-negative bacteria, fungi, viruses, parasites	10
LAMP	Antimicrobial, Antifungal, Anticancer, Antiparasitic	Natural & Synthetical	AMP Databases, UniProt, Literature,	5547	2013	Present	Absent	Absent	Absent	Gram-positive and Gram-negative bacteria, fungi, viruses, parasites	11
DBAASP v3	Antibacterial, antifungal, antiviral, anticancer, and antitumour	Natural & Synthetical	Google and PubMed, PDB	>15 700	2021	Present	Absent	Present	Absent	Gram-positive, Gram-negative, virus, parasite, insect, cancer, fungus, mammalian cell and mycoplasma	12
dbAMP 2.0	Antimicrobial, antibacterial, antiGram positive bacterial, anti-Gram negative bacterial, antifungal, antiviral, antiparasitic	Natural & Synthetical	NCBI, UniProt, Protein Data Bank, AMPs Databases	26, 447	2022	Present	Absent	Absent	Absent	Gram-positive and Gram-negative bacteria, fungi, viruses, parasites	13
AVPdb	Virus, viral, peptide, inhibit, block	Natural & Synthetical	Pubmed, AMPs Databases	2683	2014	Absent	Absent	Absent	Absent	viruses	14
DRAMP 3	AMPs, host defence peptides, antibacterial peptides, antifungal peptides, antiviral peptides, anticancer peptides, etc	Natural, synthetic, patent, and clinical AMPs	PubMed, Web of Science, Lens, ClinicalTrials.gov, chinadrugtrials.org.cn, Literature, UniProt, PDB,	22 259	2021	Absent	Absent	Absent	Absent	Gram-positive, Gram-negative, virus, parasite, insect, fungus, mammalian cell, mycoplasma, and protozoa	15
ADAPTABLE	–	Predicted or experimental	Pubmed, AMPs Databases	more than 40 000	2019	Absent	Absent	Absent	Absent	Gram-positive and Gram-negative bacteria, fungi, viruses, parasites	16

In comparison to the existing databases, AbAMPdb distinguishes itself by offering novel features that enhance its utility. Unlike other databases, it provides 3D modelled structures for all the extracted natural and synthetic (mutated) AMPs, a unique feature that is not commonly found elsewhere. Furthermore, AbAMPdb provides AMP-target protein interaction (docking) data, a valuable resource not provided by other databases. This concept accords with the past data-based research that has drawn a deep correlation between the physicochemical properties of AMPs and their docking profiles, facilitating deeper insights into AMP mechanisms, and supporting further research in antimicrobial therapy development.

The comparison between AbAMPdb and other databases highlights its unique features. Specifically tailored to curate *A. baumannii*-specific AMPs, AbAMPdb incorporates various physicochemical descriptors to accurately evaluate AMP activity, regardless of their antibacterial properties. This focused approach reflects the recognition of the importance of addressing the specific challenges posed by *A. baumannii* infections. However, it is important to note that the structures of all AMPs included in AbAMPdb are not readily accessible on widely used platforms like UniProt and the Protein Data Bank. This limitation may present challenges for researchers seeking to employ these structures in their *in silico* approaches, indicating a potential area for future development or collaboration to enhance data accessibility and usability.

## Conclusion

AbAMPdb is a manually curated database of AMPs designed specifically against *A. baumannii*. AbAMPdb holds 300 AMPs, categorized as 250 natural AMPs and 50 synthetic or mutant AMPs with distinct amino acid substitutions. AbAMPdb encompasses 5000 docked complexes complemented by 5000 images illustrating 2D visualization of interacting residues.

However, by elucidating the relationship between AMP sequences and structure against *A. baumannii* pathogenic proteins can be better understood to design new therapeutics against *A. baumannii*. The continual expansion of AbAMPdb by adding newly discovered AMPs against *A. baumannii* will further enhance the utility and significance of the database. AbAMPdb may serve as knowledgeable resource to expedite the *in vitro* and *in vivo* studies by limiting the time and resources needed to accomplish the design and development of AMP-based therapeutics.

## Supplementary Material

baae096_Supp

## Data availibility

AbAMPdb is publicly available at https://abampdb.mgbio.tech/.
