# Supplementary material for: AbAMPdb: a database of Acinetobacter baumannii specific antimicrobial peptides
Source: Database (Oxford). 2024 Oct 12;2024:baae096. doi: 10.1093/database/baae096 (PMC11470754; doi:10.1093/database/baae096)
Supplement: baae096_Supp [file baae096_supp.zip › suppl_data/S1-Supporting Information File.docx]

|  | **Table S1.** Experimental AMPs and target proteins collected from literature. Experimental AMPs screened from literature against *A. baumannii* and antibiotic resistance proteins and virulence factors involved in *A. baumannii* .pathogenesis selected from literature. | | | | | |
| --- | --- | --- | --- | --- | --- | --- |
| **AMP_Name** | | **Sequence** | **PDB entries** | **Reference** | **Target Proteins** | **Reference** |
| Aurein 1.2 | | GLFDIIKKIAESF | 1VM5 | **(Jaśkiewicz et al. 2019)** | OMP_33-36 | (Rumbo et al. 2014) |
| CAMEL | | KWKLFKKIGAVLKVL | 2JMY | **(Jaśkiewicz et al. 2019)** | OMPW | (Huang et al. 2015) |
| Citropin 1.1 | | GLFDVIKKVASVIGGL | P81835 | **(Jaśkiewicz et al. 2019)** | fimH | (Padmaja et al. 2020) |
| LL-37 | | LLGDFFRKSKEKIGKEFKRIVQRIKDFLRNLVPRTES | 2K6O | **(Jaśkiewicz et al. 2019)** | LptD | (Bojkovic et al. 2016) |
| Omiganan | | ILRWPWWPWRRK | Nil | **(Jaśkiewicz et al. 2019)** | FBN | (Smani et al. 2012) |
| r-Omiganan | | KRRWPWWPWRLI | Nil | **(Jaśkiewicz et al. 2019)** | cat-1 | (Tavakol et al. 2018) |
| Pexiganan | | GIGKFLKKAKKFGKAFVKILKK | Nil | **(Jaśkiewicz et al. 2019)** | oprD | (Dupont et al. 2005) |
| Temporin A | | FLPLIGRVLSGIL | 2MAA | **(Jaśkiewicz et al. 2019)** | OXA_51_like | (Brown et al. 2005) |
| Cec4 | | GWLKKIGKKIERVGQNTRDATIQAIGVAQQAANVAATLKG | Nil | (Peng et al. 2019) | adeA | (Lopes and Amyes 2013) |
| Cec4-7 | | GWLKKIGKKIERVGQHTRDATIQAIGVAQQAANVAATLKG | Nil | (Peng et al. 2019) | adeC | (Lopes and Amyes 2013) |
| Cec4-8 | | GWVKKIGKKIERVGQNTRDATIQVIGVAQQAANVAATLKG | Nil | (Peng et al. 2019) | adeR | (Lopes and Amyes 2013) |
| Agelaia-MPI | | INWLKLGKAIIDAL | Nil |  | adeS | (Lopes and Amyes 2013) |
| Human KS-30 (Cathelicidin) | | KSKEKIGKEFKRIVQRIKDFLRNLVPRTES | 2K6O | (Murakami et al. 2004) | ampC | (Liu and Liu 2015) |
| Human KR-20 (Cathelicidin) | | KRIVQRIKDFLRNLVPRTES | 2K6O | (Murakami et al. 2004) | baeR | (Lin et al. 2014) |
| KR-12 ( Cathelicidin) | | KRIVQRIKDFLR | Nil | (Wang 2008) | baeS | (Lin et al. 2014) |
| SAAP-148 | | LKRVWKRVFKLLKRYWRQLKKPVR | Nil | (van Gent et al. 2022) | bfmR | (Krasauskas et al. 2019) |
| AM-CATH36 | | GLFKKLRRKIKKGFKKIFKRLPPIGVGVSIPLAGKR | Nil | (Neshani et al. 2020) | smpA | (Li et al. 2017) |
| AM-CATH28 | | KIKKGFKKIFKRLPPIGVGVSIPLAGKR | Nil | (Neshani et al. 2020) | cm1A | (Taitt et al. 2014) |
| AM-CATH21 | | GLFKKLRRKIKKGFKKIFKRL | Nil | (Neshani et al. 2020) | strA | (Cherubini et al. 2022) |
| Cathelicidin-BF | | KFFRKLKKSVKKRAKEFFKKPRVIGVSIPF | Nil | (Neshani et al. 2020) | sul1 | (Tavakol et al. 2018) |
| NA-CATH | | KRFKKFFKKLKNSVKKRAKKFFKKPKVIGVTFPF | Nil | (Neshani et al. 2020) |  |  |
| WAM1 | | KRGFGKKLRKRLKKFRNSIKKRLKNFNVVIPIPLPG | Nil | (Neshani et al. 2020) |  |  |
| Bactenecin | | RLCRIVVIRVCR | P22226 | (Neshani et al. 2020) |  |  |
| Indolicidin | | ILPWKWPWWPWRR | 1G89 | (Neshani et al. 2020) |  |  |
| HNP‐1 | | ACYCRIPACIAGERRYGTCIYQGRLWAFCC | 3GNY | (Neshani et al. 2020) |  |  |
| HNP‐2 | | CYCRIPACIAGERRYGTCIYQGRLWAFCC | 3GNY | (Neshani et al. 2020) |  |  |
| HD5d5 | | ARARCRRGRAARRRRLRGVCRIRGRLRRLAAR | Nil | (Neshani et al. 2020) |  |  |
| HBD-2 | | GIGDPVTCLKSGAICHPVFCPRRYKQIGTCGLPGTKCCKKP | 1FD3 | (Neshani et al. 2020) |  |  |
| HBD-3 | | GIINTLQKYYCRVRGGRCAVLSCLPKEEQIGKCSTRGRKCCRRKK | 1KJ6 | (Neshani et al. 2020) |  |  |
| Magainin-2 | | GIGKFLHSAKKFGKAFVGEIMNS | 2MAG | (Neshani et al. 2020) |  |  |

| (P)PAP-A3, P-Pepsin A-3 (16-40)-P-Pepsin A-3 (41-61) | PIMYKVPLIRKKSLRRTLSERGLLKDPFLKKHNLNPARKYFPQWKAPTL | | DBAASP | | Nil | |
| --- | --- | --- | --- | --- | --- | --- |
| Aa Cecropin A, Aa Cecropin A2 | GGLKKLGKKLEGAGKRVFNAAEKALPVVAGAKALRK | | (Rangel et al. 2023) | | Nil | |
| AAEL000598-PA, Aedesin | GGLKKLGKKLEGAGKRVFKASEKALPVVVGIKAIGK | | DBAASP | | Nil | |
| Aborycin | CLGIGSCNDFAGCGYAVVCFW | | DBAASP | | 1RPB | |
| Anoplin | GLLKRIKTLL | | DBAASP | | 2MJQ | |
| Arenicin-1 | RWCVYAYVRVRGVLVRYRRCW | | DBAASP | | 2jsb | |
| As Cathelicidin 4, As-CATH4 | RRGLFKKLRRKIKKGFKKIFKRLPPVGVGVSIPLAGRR | | DBAASP | | Nil | |
| As Cathelicidin 5, As-CATH5 | TRRKFWKKVLNGALKIAPFLLG | | DBAASP | | Nil | |
| As Cathelicidin 6, As-CATH6 | TRWLWLLRGGLKAAGWGIRAHLNRNQ | | DBAASP | | Nil | |
| Astacidin PcAst-1a | SNGYRPAYRPAYRPSYRP | | DBAASP | | Nil | |
| Astacidin PcAst-1b/c | SNVYRPPPYRPVYRPLRRPGYRP | | DBAASP | | Nil | |
| Astacidin PcAst-2 | FYPRPYRPPYLPDPRPFPRPLPAFGHEFRRH | | DBAASP | | Nil | |
| Bicarinalin | KIKIPWGKVKDFLVGGMKAV | | (Rangel et al. 2023) | | Nil | |
| Bombinin-H2 | IIGPVLGLVGSALGGLLKKI | | DBAASP | | P82285 | |
| Bombinin-H5 | IGPVLGLVGSALGGLLKKI | | DBAASP | | P82285 | |
| BP100 | KKLFKKILKYL | | (Rangel et al. 2023) | | Nil | |
| BP202 | KRLFRKILKYL | | (Majumder et al. 2019) | | Nil | |
| BP214 | KKLFKKILRYL | | (Rangel et al. 2023) | | Nil | |
| BR001 | KWKLFKKIEKVGQNIRDGIIKAGPAVAVVGQATQIAK | | (Rangel et al. 2023) | | P01507 | |
| BR002 | GWLKKIGKKIERVGQHTRDATIQGLGIAQQAANVAATAR | | (Majumder et al. 2019) | | P08375 | |
|  |  | |  | |  | |
| aHylin a1-15K | IAKAILPLALKALKKLIK | | (Rangel et al. 2023) | | Nil | |
|  |  |  |  |  |  | |
| BR005 | RGFRKHFNKLVKKVKHTISETAHVAKDTAVIAGSGAAVVAAT | | (Majumder et al. 2019) | | 1ZRX | |
|  |  |  |  | |  | |
| BR029 | KWKIFKKIEKAGRNIRDGIIKAGPAVSVVGEAATIYKTG | | (Majumder et al. 2019) | | Nil | |
|  |  | |  | |  | |
| BR030 | KWKFYKKIERVGQNIRDGIIKAGPAVQVVGQQPRYIKENRFYS | | (Majumder et al. 2019) | | Nil | |
|  |  | |  | |  | |
| BR031 | GWLRDFGKRIERVGQHTRDATIQAIGVAQQAANVAATVRG | | (Majumder et al. 2019) | | Nil | |
| BR032 | GWLKKIGKKIERVGQHTRDATIQVLGVAQQAANVGPATARG | | (Majumder et al. 2019) | | Nil | |
| BR033 | GWLKKIGKKIERVGQHTRDATIQTIGVAQQAANVAATLKG | | (Rangel et al. 2023) | | Nil | |
| BR034 | GWLKKFGKKIERVGQHTRDATIQAIGVAQQAANVAATLKG | | (Majumder et al. 2019) | | Nil | |
| BR035 | GWLKKIGKKIERVGQHTRDASIQAIGIAQQAANVAATARG | | (Majumder et al. 2019) | | Nil | |
| BR036 | GWLKKIGKKIERVGQHTRDATIQVLGVAQQAANVAATARG | | (Majumder et al. 2019) | | Nil | |
| BR037 | GLVKKIGKKIERVGQHTRDASIQAIGIAQQAANVAATARG | | (Majumder et al. 2019) | | Nil | |
| BR043 | AGFRKRFNKLVKKVKHTIKETANVSKDVAIVAGSGVAVGAAMG | | (Rangel et al. 2023) | | Nil | |
| BR044 | GFRKRFNKLVKKVKHTIKETANVSKDVAIVAGSGVAVGAAMG | | (Majumder et al. 2019) | | Nil | |
| Bthepc | QSHLSLCRWCCNCCHNKGCGFCCKF | | DBAASP | | Nil | |
| C18G | ALWKKLLKKLLKSAKKLG | | (Majumder et al. 2019) | | Nil | |
| C18G-Arg | ALWRRLLRRLLRSARRLG | | (Majumder et al. 2019) | | Nil | |
| C18G-His | ALWHHLLHHLLHSAHHLG | | (Majumder et al. 2019) | | Nil | |
| C20 | KKIMRTFLRRISKDILTGKK | | (Majumder et al. 2019) | | 1NKL | |
| C20-DK | KKIMRTFLRRISKKILTGKK | | (Majumder et al. 2019) | | Nil | |
| CATH_BRALE | RRSKARGGSRGSKMGRKDSKGGSRGRPGSGSRPGGGSSIAGASRGDRGGTRNA | | DBAASP | | Nil | |
| Cathelicidin 4, buCATHL4B | AIPWIWIWRLLRKG | | DBAASP | | Nil | |
| α-Helical-26 (A12L/A20L) | KWKSFLKTFKSLKKTVLHTLLKAISS | | (Rangel et al. 2023) | | Nil | |
| Cathelicidin CATH2, Cc-CATH2 | LVQRGRFGRFLKKVRRFIPKVIIAAQIGSRFG | | DBAASP | | Nil | |
| Cathelicidin CATH3, Cc-CATH3 | RVRRFWPLVPVAINTVAAGINLYKAIRRK | | DBAASP | | Nil | |
| Cathelicidin CATH3, Pc-CATH1 | RIKRFWPVVIRTVVAGYNLYRAIKKK | | DBAASP | | Nil | |
| Cathelicidin Ps-CATH1 | RRSRSPRRKWTWKPRRRGSYTLISQGGNKGKH | | DBAASP | | Nil | |
| Cathelicidin Ps-CATH3 | TRSRWRRFTRRAGGFIRKNRWNIISTALKWIG | | DBAASP | | Nil | |
| Cathelicidin Ps-CATH4 | TRGRWGRFKRRAGRFIRRNRWQIISTGLKLIG | | DBAASP | | Nil | |
| Cathelicidin Ps-CATH5 | ASLLSLALMSQTTPIHPTPNYRTAWFLFSGLLGSAGVLFFLEAPTEVRSGG | | DBAASP | | Nil | |
| Cathelicidin Ps-CATH6 | KKPSKKPKPQAMTFPKVTVEYFPASFSTAALTVPED | | DBAASP | | Nil | |
| Am23SK | SCRFSGGYCIWNWERCRSGHFLVALCPFRKRCCK | | (Rangel et al. 2023) | | Nil | |
| Bactenecin | LCRIVVIRVCR | | (Rangel et al. 2023) | | Nil | |
| Cathelicidin, EA-CATH1 | KRRGSVTTRYQFLMIHLLRPKKLFA | | DBAASP | | Nil | |
| Cathelicidin-3.4, ChBac3.4 | RFRLPFRRPPIRIHPPPFYPPFRRFL | | DBAASP | | Nil | |
| Cathelicidin-5, myeloid antimicrobial peptide BMAP-28 | | GGLRSLGRKILRAWKKYGPIIVPIIRI | | DBAASP | | P54229 |
| Cathelicidin-like peptide, Batroxicidin | | KRFKKFFKKLKNSVKKRVKKFFRKPRVIGVTFPF | | DBAASP | | Nil |
| Cathelicidin-like peptide, Crotalicidin | | KRFKKFFKKVKKSVKKRLKKIFKKPMVIGVTIPF | | DBAASP | | 2MWT |
| Cathelicidin-OH antimicrobial peptide, Oh_CRAMP | | KRFKKFFKKLKNSVKKRAKKFFKKPRVIGVSIPF | | (Rangel et al. 2023) | | Nil |
| Cathelicidin-RC1 | | KKCKFFCKVKKKIKSIGFQIPIVSIPFK | | DBAASP | | Nil |
| Cathelicidin-RC2 | | KKCGFFCKLKNKLKSTGSRSNIAAGTHGGTFRV | | DBAASP | | Nil |
| Cathelicidin-related antimicrobial peptide isoform, Pt_CRAMP1 | | KRFKKFFMKLKKSVKKRVMKFFKKPMVIGVTFPF | | DBAASP | | Nil |
| BmKn1 | | FIGAVAGLLSKIF | | (Rangel et al. 2023) | | Nil |
| BR003-cecropin A | | GGLKKLGKKLEGAGKRVFNAAEKALPVVAGAKALRK | | (Majumder et al. 2019) | | Nil |
| Cecropin Sb, SibaCec | | GKLTKDKLKRGAKKALNVASKVAPIVAAGASIAR | | DBAASP | | Nil |
| Cecropin-B, Cecropin XJ | | RWKIFKKIEKMGRNIRDGIVKAGPAIEVLGSAKAI | | DBAASP | | P04142 |
| CecropinXJ | | RWKIFKKIEKMGRNIRDGIVKAGPAIEVLGSAKAIGK | | DBAASP | | Nil |
| CL-Defensin | | ATCDLFSFQSKWVTPNHAACAAHCTARGNRGGRCKKAVCHCRK | | DBAASP | | Nil |
| CLP-19 | | CRKPTFRRLKWKIKFKFKC | | (Majumder et al. 2019) | | Nil |
| CMB001 | | WKSQFCPGCVGVLQCFIQACNCHIK | | (Karczewski et al. 2020) | | Nil |
| CMB001 | | WKQFPGVGVLQFIQANAHIK | | (Karczewski et al. 2020) | | Nil |
| Cm-CATH1 | | RRSIFRKLRRKIKKGLKKGIQHLLAGGRQGLPQGGRPGMI | | DBAASP | | Nil |
| Cm-CATH2 | | RRSRFGRFFKKVRKQLGRVLRHSRITVGGRMRF | | DBAASP | | Nil |
| Cm-CATH3 | | TRGRWKRFWRGAGRFFRRHKEKIIRAAVDIVLS | | DBAASP | | Nil |
| Cm-CATH4 | | MAFPFSTQRINPEIEEGNASLADLPVTHAGSLPGIKAQVRTALGIALLLVA | | DBAASP | | Nil |
| CopW | | LLWIALRKK | | DBAASP | | Nil |
| Ctriporin | | FLWGLIPGAISAVTSLIKK | | (Rangel et al. 2023) | | Nil |
| Cu Hepcidin, CuHepc | | QSHLSLCRWCCNCCHNKSCGFCCKF | | DBAASP | | Nil |
| Defensin Mdde, Phormicin C | | ATCDLLSGTGVGHSACAAHCLLRGNRGGYCNGKGVCVCRN | | DBAASP | | Nil |
| Defensin, isoform C | | FTCDVLGFEIAGTKLNSAACGAHCLALGRTGGYCNSKSVCVCR | | DBAASP | | Nil |
| Dicentracin-like peptide | | FLRSLLRGAKAIYRGARAGWRG | | DBAASP | | Nil |
| Distinctin chain 1 | | ENREVPPGFTALIKTLRKCKII | | DBAASP | | Nil |
| Distinctin chain 2 | | NLVSGLIEARKYLEQLHRKLKNCKV | | DBAASP | | 1XKM |
| Dybowskin-2CDYa, Chensinin-1 | | SAVGRHGRRFGLRKHRKH | | DBAASP | | Nil |
| Buforin II | | TRSSRAGLQFPVGRVHRLLRK | | (Rangel et al. 2023) | | Nil |
| Esculentin-2CHa | | GFSSIFRGVAKFASKGLGKDLAKLGVDLVACKISKQC | | DBAASP | | Nil |
| Frenatin 2D | | DLLGTLGNLPLPFI | | DBAASP | | Nil |
| Gramicidin S, GS | | VLfPVLfP | | DBAASP | | Nil |
| H4 | | KFKKLFKKLSPVIGKEFKRIVERIKRFLR | | (Majumder et al. 2019) | | Nil |
| Hainanenin 1 | | FALGAVTKLLPSLLCMITRKC | | DBAASP | | Nil |
| Hainanenin 5 | | FALGAVTKRLPSLFCLITRKC | | DBAASP | | Nil |
| Caerin 1.1 | | GLLSVLGSVAKHVLPHVVPVIAEHL | | (Rangel et al. 2023) | | Nil |
| Hc-CATH | | KFFKRLLKSVRRAVKKFRKKPRLIGLSTLL | | DBAASP | | Nil |
| Hepcidin-20 | | ICIFCCGCCHRSKCGMCCKT | | DBAASP | | 1M4E |
| HFIAP-1 | | GFFKKAWRKVKHAGRRVLDTAKGVGRHYVNNWLNRYRZ | | DBAASP | | Nil |
| HFIAP-3 | | GWFKKAWRKVKNAGRRVLKGVGIHYGVGLI | | DBAASP | | Nil |
| Hp1404 | | GILGKLWEGVKSIF | | (Rangel et al. 2023) | | Nil |
| H-Tridecaptin B1 | | GGswSIEVA | | DBAASP | | Nil |
| Human beta-defensin-4, hBD4 | | EFELDRICGYGTARCRKKCRSQEYRIGRCPNTYACCLRKWDESLLNRTKP | | DBAASP | | Nil |
| Caerin 1.9 | | GLFGVLGSIAKHVLPHVVPVIAEKL | | (Rangel et al. 2023) | | 2K6O |
| Hydramacin-1 | | QIVDCWETWSRCTKWSQGGTGTLWKSCNDRCKELGRKRGQCEEKPSRCPLSKKAWTCICY | | DBAASP | | 2K35 |
| Hylin-a1 | | IFGAILPLALGALKNLIK | | (Rangel et al. 2023) | | 2N0O |
| I10 | | KKIMRTFLRR | | (Majumder et al. 2019) | | 1NKL |
| Im-5 | | FLGSLFSIGSKLLPGVIKLFQRKKQ | | (Rangel et al. 2023) | | Nil |
| Japonicin-1Npa | | FLLFPLMCKIQGKC | | DBAASP | | Nil |
| Japonicin-1Npb | | FVLPLVMCKILRKC | | DBAASP | | Nil |
| Lantibiotic NAI-107, Microbisporicin | | VSLCPGCTSGGGSNCSFC | | DBAASP | | Nil |
| Lantibiotic Nisin-A | | IILPGKGALMGNMKAHSIHVK | | DBAASP | | Nil |
| Lantibiotic paenibacillin, M152-P4 | | AIIKIKVKAVCKLCICGCNCK | | DBAASP | | Nil |
| Latarcin-3a, Ltc-3a | | SWKSMAKKLKEYMEKLKQRA | | DBAASP | | Nil |
| LS-Sarcotoxin, MD-Cecropin | | GWLKKIGKKIERVGQHTRDATIQTIGVAQQAANVAATLK | | DBAASP | | Q06590 |
| LS-stomoxyn | | GFRKRFNKLVKKVKHTIKETANVSKDVAIVAGSGVAVGAAM | | (Rangel et al. 2023) | | Nil |
| Lycosin-II | | VWLSALKFIGKHLAKHQLSKL | | DBAASP | | Nil |
| LyeTx 1 | | IWLTALKFLGKNLGKHLAKQQLAKL | | DBAASP | | Nil |
| LysAB2 P0 | | NPEKALEPLIAIQIAIKGMLNGWFTGVGFRRKR | | (Majumder et al. 2019) | | Nil |
| LysAB2 P1 | | EKALEKLIAIQKAIKGMLNGWFTGVGFRRKR | | (Majumder et al. 2019) | | Nil |
| LysAB2 P2 | | EKALEKLIAIQKAIKGMLAGWFTGVGARRKR | | (Majumder et al. 2019) | | Nil |
| LysAB2 P3 | | NPEKALEKLIAIQKAIKGMLNGWFTGVGFRRKR | | (Majumder et al. 2019) | | Nil |
| Mastoparan B | | LKLKSIVSWAKKVL | | DBAASP | | P21564 |
| Mastoparan-L | | INLKALAALAKKIL | | (Rangel et al. 2023) | | 1D7N |
| Mastoparan-M, Mast cell-degranulating peptide, Mastoparan-like peptide 12c | | INLKAIAALAKKLL | | DBAASP | | P04205 |
| Medipeptin A | | PAAAVVTVIBGAAVTAOSI | | DBAASP | | Nil |
| Mini-ChBac7.5N alpha | | RRLRPRRPRLPRPRPRPRPRPR | | (Rangel et al. 2023) | | Nil |
| Mini-ChBac7.5N beta | | RRLRPRRPRLPRPRPRPRPRP | | (Rangel et al. 2023) | | Nil |
| M-myrmeciitoxin-Mp2a | | IDWKKVDWKKVSKKTCKVMLKACKFL | |  | | Nil |
| Moronecidin-like peptide | | FFRNLWKGAKAAFRAGHAAWRA | | 6.25 | | Nil |
| M-poneratoxin-Dq3a, Dq-2562 | | FWGTLAKWALKAIPAAMGMKQNK | |  | | Nil |
| N17 | | KILRGVSKKIMRTFLRR | | (Majumder et al. 2019) | | Nil |
| Natto peptide | | SMATPHVAGAAALILSKHPTWTNAQVRDRLESTATYLGNSFYYGK | | 128 | | Nil |
| Neutrophil defensin 3, HNP-3 | | DCYCRIPACIAGERRYGTCIYQGRLWAFCC | |  | | 1DFN |
| Neutrophil defensin 4, Defensin alpha 4, HNP-4 | | VCSCRLVFCRRTELRVGNCLIGGVSFTYCCTRV | | 0.8 | | 1ZMM |
| Nicomicin-1 | | GFWSSVWDGAKNVGTAIIKNAKVCVYAVCVSHK | |  | | 6HN9 |
| NK10 | | ISKRILTGKK | | (Majumder et al. 2019) | | Nil |
| NK11 | | KISKRILTGKK | | (Majumder et al. 2019) | | Nil |
| NK13 | | KISKKIMRTFLRR | | (Majumder et al. 2019) | | Nil |
| NK14 | | KILGVSKRILTGKK | | (Majumder et al. 2019) | | Nil |
| NK15 | | KILRGVSKRILTGKK | | (Majumder et al. 2019) | | Nil |
| NK19a | | KISKKIMRTFLRRILTGKK | | (Majumder et al. 2019) | | Nil |
| NK19b | | KILRGVSKKIMRRILTGKK | | (Majumder et al. 2019) | | Nil |
| NK19b-KR | | RILRGVSRRIMRRILTGRR | | (Majumder et al. 2019) | | Nil |
| NK-2 | | KILRGVCKKIMRTFLRRISKDILTGKK | | (Majumder et al. 2019) | | 1NKL |
| NK22b | | KILGVSKKIMRRISKDILTGKK | | (Majumder et al. 2019) | | Nil |
| NK23a | | KISKKIMRTFLRRISKDILTGKK | | (Majumder et al. 2019) | | Nil |
| NK23b | | KILRGVSKKIMRRISKDILTGKK | | (Majumder et al. 2019) | | Nil |
| NK23c | | KILRGVSKKIMRTFLRRILTGKK | | (Majumder et al. 2019) | | Nil |
| NK27 | | KILRGVSKKIMRTFLRRISKDILTGKK | | (Majumder et al. 2019) | | Nil |
| PaDBS1R1 | | PKILNKILGKILRLAAAFK | | DBAASP | | Nil |
| Parkerin | | GWANTLKNVAGGLCKITGAA | | DBAASP | | Nil |
| Caerin 1.1 + Caerin 1.9 | | GLLSVLGSVAKHVLPHVVPVIAEHLGLFGVLGSIAKHVLPHVVPVIAEKL | | (Rangel et al. 2023) | | Nil |
| Pepcon | | FLFSLIPSAIGGLISAFK | | (Majumder et al. 2019) | | Nil |
| Peptide BmKn2 | | FIGAIARLLSKIF | | (Rangel et al. 2023) | | Nil |
| Phormicin D | | ATCDLLSAYKVAHSACAAHCLLRGNRGGYCNSRAVCVCRN | | DBAASP | | Nil |
| Phylloseptin-S2, PLS-S2 | | FLSLIPHIVSGVASLAKHF | | DBAASP | | Nil |
| Phylloseptin-S3, PLS-S3 | | FLSLIPHIVSGVASLAIHF | | DBAASP | | Nil |
| Phylloseptin-S4, PLS-S4 | | FLSMIPHIVSGVAALAKHL | | DBAASP | | Nil |
| Piscidin 1, Moronecidin | | FFHHIFRGIVHVGKTIHRLVTG | | (Rangel et al. 2023) | | 2jos |
| PN-CATH1 | | KKCNFFCKLKKKVKSVGSRNLIGSATHHHRIYRV | | DBAASP | | Nil |
| PN-CATH2 | | EGCNILCLLKRKVKAVKNVVKNVVKSVVG | | DBAASP | | Nil |
| Prepromelittin-related peptide, MRP, AR-23 | | AIGSILGALAKGLPTLISWIKNR | | DBAASP | | Nil |
| Protegrin 1, PG-1, Neutrophil peptide 1 | | RGGRLCYCRRRFCVCVGR | | (Rangel et al. 2023) | | 1PG1 |
| Ps Hepcidin | | QSHLSLCRYCCNCCRNKGCGYCCKF | | DBAASP | | Nil |
| Pseudhymenochirin-1Pb [R8r] | | IKIPSFFRNILKKVGKEAVSLIAGALKQS | | DBAASP | | Nil |
| Ranalexin | | LGGLIKIVPAMICAVTKKC | | (Rangel et al. 2023) | | P39084 |
| rr | | WLRRIKAWLRR | | (Rangel et al. 2023) | | 7YSS |
| rr1 | | WKRRIKIWKKIR | | (Majumder et al. 2019) | | Nil |
| rr2 | | WIRRIKKWIRRVHK | | (Rangel et al. 2023) | | Nil |
| rr3 | | WLRRIKAWLRRKRK | | (Majumder et al. 2019) | | Nil |
| rr4 | | WLRRIKAWLRRIKA | | (Rangel et al. 2023) | | 7YSS |
| RW-BP10 | | RRLFRRILRWL | | (Majumder et al. 2019) | | Nil |
| Smp43(1-14) | | GVWDWIKKTAGKIW | | DBAASP | | Nil |
| Sparamosin26-54 | | GLGPNPCRKKCYKRDFLGRCRLNFTCMFG | | DBAASP | | Nil |
| TA1686 Hepcidin-25 / LEAP | | DTHFPICIFCCGCCHRSKCGMCCKT | | DBAASP | | 2KEF |
| Tachyplesin-1 | | KWCFRVCYRGICYRRCR | | DBAASP | | 1WO1 |
| Temporin-PTa | | FFGSVLKLIPKIL | | DBAASP | | Nil |
| Temporin-SHd | | FLPAALAGIGGILGKLF | | DBAASP | | Nil |
| Temporin-SHe | | FLPALAGIAGLLGKIF | | DBAASP | | Nil |
| Tilapia piscidin 1, TP1 | | FDWDSVLKGVEGFVRGYF | | DBAASP | | Nil |
| Tilapia piscidin 2, TP2 | | GECIWDAIFHGAKHFLHRLVNP | | DBAASP | | Nil |
| Tremporin-1Cec | | IIPLPLGYFAKKT | | DBAASP | | Nil |
| Trichoplaxin | | FFGRLKSVWSAVKHGWKAAKSR | | DBAASP | | Nil |
| VsCT1 | | FLKGIIDTVSNWL | | (Rangel et al. 2023) | | Nil |
| Xac-1, Xylopin | | GFVALLKKLPLILKHLH | | DBAASP | | Nil |
| CAME | | KWKLFKKIGIGAVLKVLTTG | | (Rangel et al. 2023) | | Nil |
| BMAP-27 | | GRFKRFRKKFKKLFKKLSPVIPLLHLG | | (Rangel et al. 2023) | | 2KET |
| GW-A2 | | GAKYAKIIYNYLKKIANALW | | (Rangel et al. 2023) | | Nil |
| SMAP-29 | | RGLRRLGRKIAHGVKKYGPTVLRIIRIAG | | (Rangel et al. 2023) | | 1FRY |
| GW-H1a | | GYNYAKKLANLAKKFANALW | | (Jung et al. 2021) | | Nil |
| Latarcin-2a | | GLFGKLIKKFGRKAISYAVKKARGKH | | (Rangel et al. 2023) | | 2G9P |
| Maximin H2 | | ILGPVLSMVGSALGGLIKKI | | (Rangel et al. 2023) | | Nil |
| NRC12 | | GWKKWFNRAKKVGKTVGGLAVDHYL | | (Rangel et al. 2023) | | Nil |
| Pilosulin | | GLGSVFGRLARILGRVIPKV | | (Rangel et al. 2023) | | Nil |
| Pleurocidin | | GWGSFFKKAAHVGKHVGKAALTHYL | | (Rangel et al. 2023) | | 1Z64 |
| GW-Q6 | | GIKIAKKAITIAKKIAKIYW | | (Rangel et al. 2023) | | Nil |
| Tilapia piscidin 4 TP4 | | FIHHIIGGLFSAGKAIHRLIRRRRR | | (Rangel et al. 2023) | | 5H2S |
| Hp1404-A | | GILGKLWEGVKSIA | | (Hong et al. 2021) | | Nil |
| Hp1404-K | | GILGKLWEGVSIK | | (Hong et al. 2021) | | Nil |
| Hp1404-V | | GILGKLWEGVKSIV | | (Hong et al. 2021) | | Nil |
| Hp1404-L | | GILGKLWEGVKSIL | | (Hong et al. 2021) | | Nil |
| Hp1404-I | | GILGKLWEGVKSII | | (Hong et al. 2021) | | Nil |
| Hp1404-W | | GILGKLWEGVKSIW | | (Hong et al. 2021) | | Nil |
| HD5 | | ATCYCRTGRCATRESLSGVCEISGRLYRLCCR | | (Laneri et al. 2021) | | 1ZMP |
| B2RP | | GIWDTIKSMGKVFAGKILQNL | | (Rangel et al. 2023) | | Nil |
| B2RP-Era | | GVIKSVLKGVAKTVALGML | | (Rangel et al. 2023) | | Nil |
| Alyteserin-1c | | GLKEIFKAGLGSLVKGIAAHVAS | | (Rangel et al. 2023) | | 2L5R |
| Alyteserin-2a | | ILGKLLSTAAKLLSNL | | (Rangel et al. 2023) | | Nil |
| PGLa-AM1 | | GMASKAGSVLGKVAKVALKAAL | | (Rangel et al. 2023) | | Nil |
| CPF-AM1 | | GLGSVLGKALKIGANLL | | (Rangel et al. 2023) | | Nil |
| CPF-B1 | | GLGSLLGKAFKIGLKTVGKMMGGAPREQ | | (Rangel et al. 2023) | | Nil |
| CPF-C1 | | GFGSLLGKALRLGANVL | | (Rangel et al. 2023) | | Nil |
| Hymenochirin-1B | | IKLSPETKDNLKKVLKGAIKGAIAVAKMV | | (Neshani et al. 2020) | | Nil |
| Hymenochirin-1Pa | | LKLSPKTKDTLKKVLKGAIKGAIAIASMA | | (Rangel et al. 2023) | | Nil |
| XT-7 | | GLLGPLLKIAAKVGSNLL | | (Rangel et al. 2023) | | Nil |
| Melittin | | GIGAVLKVLTTGLPALISWIKRKRQQ | | (Rangel et al. 2023) | | 2MLT |
| Cecropin P1 | | SWLSKTAKKLENSAKKRISEGIAIAIQGGPR | | (Rangel et al. 2023) | | P14661 |
| Histatin-8 | | KFHEKHHSHRGY | | (Rangel et al. 2023) | | P34084 |
| Tachyplesin III | | KWCFRVCYRGICYRKCR | | (Rangel et al. 2023) | | 6PI3 |
| Cecropin B | | KWKIFKKIEKVGRNIRNGIIKAGPAVAVLGEAKAL | | (Majumder et al. 2019) | | P01509 |
| Magainin 1 | | GIGKFLHSAGKFGKAFVGEIMKS | | (Rangel et al. 2023) | | Nil |
| beta-Defensin | | DHYNCVSSGGQCLYSACPIFTKIQGTCYRGKAKCCK | | (Majumder et al. 2019) | | 1E4S |
| Buforin 1 | | AGRGKQGGKVRAKAKTRSSRAGLQFPVGRVHRLLRKGNY | | (Majumder et al. 2019) | | P55897 |
| Histatin 5 | | DSHAKRHHGYKRKFHEKHHSHRGY | | (Majumder et al. 2019) | | P15516 |

**References:**

Bojkovic J, Richie DL, Six DA, Rath CM, Sawyer WS, Hu Q, Dean CR (2016) Characterization of an acinetobacter baumannii lptd deletion strain: Permeability defects and response to inhibition of lipopolysaccharide and fatty acid biosynthesis. Journal of bacteriology. 198(4):731-741.

Brown S, Young H, Amyes S (2005) Characterisation of oxa-51, a novel class d carbapenemase found in genetically unrelated clinical strains of acinetobacter baumannii from argentina. Clin Microbiol Infect. 11(1):15-23.

Cherubini S, Perilli M, Segatore B, Fazii P, Parruti G, Frattari A, Amicosante G, Piccirilli A (2022) Whole-genome sequencing of st2 a. Baumannii causing bloodstream infections in covid-19 patients. Antibiotics. 11(7):955.

Dupont M, Pagès J-M, Lafitte D, Siroy A, Bollet C (2005) Identification of an oprd homologue in acinetobacter b aumannii. J Proteome Res. 4(6):2386-2390.

Huang W, Wang S, Yao Y, Xia Y, Yang X, Long Q, Sun W, Liu C, Li Y, Ma Y (2015) Ompw is a potential target for eliciting protective immunity against acinetobacter baumannii infections. Vaccine. 33(36):4479-4485.

Jaśkiewicz M, Neubauer D, Kazor K, Bartoszewska S, Kamysz W (2019) Antimicrobial activity of selected antimicrobial peptides against planktonic culture and biofilm of acinetobacter baumannii. Probiotics and antimicrobial proteins. 11:317-324.

Krasauskas R, Skerniškytė J, Armalytė J, Sužiedėlienė E (2019) The role of acinetobacter baumannii response regulator bfmr in pellicle formation and competitiveness via contact-dependent inhibition system. BMC Microbiol. 19(1):1-12.

Li H, Tan H, Hu Y, Pan P, Su X, Hu C (2017) Small protein a and phospholipase d immunization serves a protective role in a mouse pneumonia model of acinetobacter baumannii infection. Mol Med Report. 16(2):1071-1078.

Lin M-F, Lin Y-Y, Yeh H-W, Lan C-Y (2014) Role of the baesr two-component system in the regulation of acinetobacter baumannii adeab genes and its correlation with tigecycline susceptibility. BMC Microbiol. 14(1):1-12.

Liu Y, Liu X (2015) Detection of ampc β-lactamases in acinetobacter baumannii in the xuzhou region and analysis of drug resistance. Exp Ther Med. 10(3):933-936.

Lopes B, Amyes S (2013) Insertion sequence disruption of ader and ciprofloxacin resistance caused by efflux pumps and gyra and parc mutations in acinetobacter baumannii. International journal of antimicrobial agents. 41(2):117-121.

Murakami M, Lopez-Garcia B, Braff M, Dorschner RA, Gallo RL (2004) Postsecretory processing generates multiple cathelicidins for enhanced topical antimicrobial defense. The Journal of Immunology. 172(5):3070-3077.

Neshani A, Sedighian H, Mirhosseini SA, Ghazvini K, Zare H, Jahangiri A (2020) Antimicrobial peptides as a promising treatment option against acinetobacter baumannii infections. Microbial pathogenesis. 146:104238.

Padmaja S, Smiline Girija A, Priyadharsini JV (2020) Frequency of adhesive virulence factor fimh among the clinical isolates of acinetobacter baumannii in india. Journal of Pharmaceutical Research International. 32(16):12-17.

Peng J, Long H, Liu W, Wu Z, Wang T, Zeng Z, Guo G, Wu J (2019) Antibacterial mechanism of peptide cec4 against acinetobacter baumannii. Infection and drug resistance.2417-2428.

Rumbo C, Tomás M, Fernandez Moreira E, Soares NC, Carvajal M, Santillana E, Beceiro A, Romero A, Bou G (2014) The acinetobacter baumannii omp33-36 porin is a virulence factor that induces apoptosis and modulates autophagy in human cells. Infection and immunity. 82(11):4666-4680.

Smani Y, McConnell MJ, Pachón J (2012) Role of fibronectin in the adhesion of acinetobacter baumannii to host cells. PloS one. 7(4):e33073.

Taitt CR, Leski TA, Stockelman MG, Craft DW, Zurawski DV, Kirkup BC, Vora GJ (2014) Antimicrobial resistance determinants in acinetobacter baumannii isolates taken from military treatment facilities. Antimicrobial agents and chemotherapy. 58(2):767-781.

Tavakol M, Momtaz H, Mohajeri P, Shokoohizadeh L, Tajbakhsh E (2018) Genotyping and distribution of putative virulence factors and antibiotic resistance genes of acinetobacter baumannii strains isolated from raw meat. Antimicrob Resist Infect Control. 7(1):1-11.

van Gent ME, van der Reijden TJ, Lennard PR, de Visser AW, Schonkeren-Ravensbergen B, Dolezal N, Cordfunke RA, Drijfhout JW, Nibbering PH (2022) Synergism between the synthetic antibacterial and antibiofilm peptide (saap)-148 and halicin. Antibiotics. 11(5):673.

Wang G (2008) Structures of human host defense cathelicidin ll-37 and its smallest antimicrobial peptide kr-12 in lipid micelles. Journal of Biological Chemistry. 283(47):32637-32643.

Rangel K, Lechuga GC, Provance Jr DW, Morel CM, De Simone SG (2023) An Update on the Therapeutic Potential of Antimicrobial Peptides against Acinetobacter baumannii Infections. Pharmaceuticals. 16(9):1281.

Hong MJ, Kim MK, Park Y (2021) Comparative antimicrobial activity of Hp404 peptide and its analogs against Acinetobacter baumannii. International journal of molecular sciences. 22(11):5540.

Jung CJ, Liao YD, Hsu CC, Huang TY, Chuang YC, Chen JW, Kuo YM, Chia JS (2021) Identification of potential therapeutic antimicrobial peptides against Acinetobacter baumannii in a mouse model of pneumonia. Scientific Reports. 11(1):7318.

Majumder A, Biswal MR, Prakash MK (2019) Computational screening of antimicrobial peptides for Acinetobacter baumannii. 14(10):e0219693.

Peng J, Long H, Liu W, Wu Z, Wang T, Zeng Z, Guo G, Wu J (2019) Antibacterial mechanism of peptide Cec4 against Acinetobacter baumannii. Infection and drug resistance. 5:2417-28.

Neshani A, Sedighian H, Mirhosseini SA, Ghazvini K, Zare H, Jahangiri A (2020) Antimicrobial peptides as a promising treatment option against Acinetobacter baumannii infections. Microbial pathogenesis. 146:104238.

Laneri S, Brancaccio M, Mennitti C, De Biasi MG, Pero ME, Pisanelli G, Scudiero O, Pero R (2021) Antimicrobial peptides and physical activity: a great hope against COVID 19. Microorganisms. 9(7):1415.

Li H, Zhang S, Du Z, Long T, Yue B (2018) The antimicrobial peptide KR-12 promotes the osteogenic differentiation of human bone marrow stem cells by stimulating BMP/SMAD signaling. RSC advances. 8(28):15547-57.

Karczewski J, Krasucki SP, Asare-Okai PN, Diehl C, Friedman A, Brown CM, Maezato Y, Streatfield SJ (2020) Isolation, Characterization and Structure Elucidation of a Novel Lantibiotic From Paenibacillus sp. Frontiers in Microbiology. 11:598789.
