# Supplementary material for: AbAMPdb: a database of Acinetobacter baumannii specific antimicrobial peptides
Source: Database (Oxford). 2024 Oct 12;2024:baae096. doi: 10.1093/database/baae096 (PMC11470754; doi:10.1093/database/baae096)
Supplement: baae096_Supp [file baae096_supp.zip › suppl_data/S2-Supporting Information File.docx]

**Table S2.** Mutant library synthesis. Amino acid substitution of Experimental AMPs to synthesize

| **Parent Name** | **Parent peptide sequence** | **Mutant peptide sequence** |  |
| --- | --- | --- | --- |
| **Pexiganan** | GIGKFLKKAKKFGKAFVKILKK | GIGKICKKAKKPGKAKIKIPKK |  |
| **Temporin A** | FLPLIGRVLSGIL | ICPPIGRKKSGIP |  |
| **Cec4** | GWLKKIGKKIERVGQNTRDATIQAIGVAQQAANVAATLKG | GKPKKIGKKICRIGQPTRIATIQAIGCAQQAAKKAATPKG |  |
|  |  |  |  |
|  |  | GPIKKIGKKICRKGQKTRPATIQAIGKAQQAACCAATIKG |  |
|  |  |  |  |
|  |  | GCKKKIGKKIPRIGQPTRKATIQAIGCAQQAAKPAATCKG |  |
|  |  | GICKKIGKKIIRCGQCTRPATIQAIGPAQQAAPCAATKKG |  |
| **Cec4-7** | GWLKKIGKKIERVGQHTRDATIQAIGVAQQAANVAATLKG | GCKKKIGKKICRVGQITRCATIQAIGKAQQAAPKAATPKG |  |
| **Cec4-8** | GWVKKIGKKIERVGQNTRDATIQVIGVAQQAANVAATLKG | GCCKKIGKKIPRKGQPTRIATIQKIGIAQQAAPIAATCKG |  |
|  |  | GKPKKIGKKIKRPGQKTRCATIQPIGCAQQAACIAATPKG |  |
|  |  |  |  |
|  |  | GKIKKIGKKIIRCGQITRPATIQIIGKAQQAAKPAATIKG |  |
|  |  |  |  |
| **FITC-Cec4** | GWLKKIGKKIERVGQNTRDATIQAIGVAQQAANVAATLKG | GPIKKIGKKICRKGQITRPATIQAIGCAQQAAKKAATIKG |  |
|  |  |  |  |
|  |  | GIPKKIGKKIKRCGQCTRKATIQAIGKAQQAACKAATPKG |  |
|  |  | GCKKKIGKKIIRCGQKTRCATIQAIGPAQQAAPPAATIKG |  |
|  |  |  |  |
|  |  | GCIKKIGKKICRPGQITRPATIQAIGCAQQAAPCAATIKG |  |
| **Human KS-30 (Cathelicidin)** | KSKEKIGKEFKRIVQRIKDFLRNLVPRTES | KSKCKIGKCKKRIIQRIKIPCRICKPRTKS |  |
|  |  | KSKKKIGKKIKRIPQRIKCIKRPCKPRTPS |  |
| **Human KR-20 (Cathelicidin)** | KRIVQRIKDFLRNLVPRTES | KRIKQRIKCKPRPICPRTIS |  |
| **KR-12 ( Cathelicidin)** | KRIVQRIKDFLR | KRIKQRIKCPKR |  |
|  |  | KRIIQRIKKIPR |  |
|  |  | KRICQRIKCKIR |  |
| **SAAP-148** | LKRVWKRVFKLLKRYWRQLKKPVR | IPRPCKRIIKPCKRKCRQCKKPKR |  |
| **AM-CATH36** | GLFKKLRRKIKKGFKKIFKRLPPIGVGVSIPLAGKR | GKCKKPRRKIKKGIKKICKRKPPIGPGISIPCAGKR |  |
| **AM-CATH28** | KIKKGFKKIFKRLPPIGVGVSIPLAGKR | KIKKGCKKIPKRKPPIGCGPSIPIAGKR |  |
|  |  | KIKKGIKKIKKRCPPIGPGCSIPKAGKR |  |
|  |  | KIKKGKKKIIKRIPPIGKGISIPPAGKR |  |
| **AM-CATH21** | GLFKKLRRKIKKGFKKIFKRL | GKCKKIRRKIKKGIKKIKKRP |  |
|  |  | GPKKKCRRKIKKGKKKIIKRC |  |
| **Cathelicidin-BF** | KFFRKLKKSVKKRAKEFFKKPRVIGVSIPF | KPIRKIKKSKKKRAKKKKPIPRCIGISIPP |  |
|  |  | KCPRKIKKSIKKRAKKKCKPPRIIGISIPC |  |
| **NA-CATH** | KRFKKFFKKLKNSVKKRAKKFFKKPKVIGVTFPF | KRCKKPIKKLKKSIKKRAKKCPKKPKIIGCTKPI |  |
|  |  | KRCKKCPKKLKPSCKKRAKKPIKKPKKIGITPPC |  |
|  |  | KRIKKKIKKLKISKKKRAKKKPKKPKCIGCTKPC |  |
| **WAM1** | KRGFGKKLRKRLKKFRNSIKKRLKNFNVVIPIPLPG | KRGIGKKIRKRCKKCRPSIKKRPKPKCPIIPIPKPG |  |
|  |  | KRGCGKKPRKRKKKPRKSIKKRKKPKIICIPIPCPG |  |
| **Bactenecin** | RLCRIVVIRVCR | RKCRIPKIRPCR |  |
| **HD5d5** | ARARCRRGRAARRRRLRGVCRIRGRLRRLAAR | ARARCRRGRAARRRRPRGCCRIRGRKRRIAAR |  |
|  |  | ARARCRRGRAARRRRCRGICRIRGRPRRKAAR |  |
| **Magainin-2** | GIGKFLHSAKKFGKAFVGEIMNS | GIGKKPCSAKKKGKAIKGCIPIS |  |
|  |  | GIGKICPSAKKPGKAPIGPIICS |  |
| **C20D1** | KKIMRTFLRRISKDILTGKK | KKICRTPLRRISKKIPTGKK |  |
|  |  | KKICRTKLRRISKPIPTGKK |  |
| **CATH_BRALE** | RRSKARGGSRGSKMGRKDSKGGSRGRPGSGSRPGGGSSIAGASRGDRGGTRNA | RRSKARGGSRGSKPGRKISKGGSRGRPGSGSRPGGGSSIAGASRGCRGGTRKA |  |
|  |  | RRSKARGGSRGSKIGRKPSKGGSRGRPGSGSRPGGGSSIAGASRGKRGGTRPA |  |
| **Cathelicidin CATH2, Cc-CATH2** | LVQRGRFGRFLKKVRRFIPKVIIAAQIGSRFG | PKQRGRIGRCCKKPRRKIPKKIIAAQIGSRPG |  |
|  |  | KPQRGRIGRCIKKPRRCIPKKIIAAQIGSRKG |  |
| **Cathelicidin CATH3, Cc-CATH3** | RVRRFWPLVPVAINTVAAGINLYKAIRRK | RPRRICPKKPPAICTVAAGICPKKAIRRK |  |
| **Cathelicidin CATH3, Pc-CATH1** | RIKRFWPVVIRTVVAGYNLYRAIKKK | RIKRCKPIPIRTKCAGIKPCRAIKKK |  |
| **Cathelicidin Ps-CATH3** | TRSRWRRFTRRAGGFIRKNRWNIISTALKWIG | TRSRKRRITRRAGGPIRKCRCKIISTAKKKIG |  |
| **Cathelicidin Ps-CATH4** | TRGRWGRFKRRAGRFIRRNRWQIISTGLKLIG | TRGRPGRKKRRAGRCIRRIRPQIISTGKKIIG |  |
|  |  | TRGRKGRKKRRAGRPIRRIRIQIISTGCKCIG |  |
