# Supplementary material for: AbAMPdb: a database of Acinetobacter baumannii specific antimicrobial peptides
Source: Database (Oxford). 2024 Oct 12;2024:baae096. doi: 10.1093/database/baae096 (PMC11470754; doi:10.1093/database/baae096)

Figure S1: Alphafold structure prediction. Alphafold structure prediction for synthetic and Natural AMPs showing pLDDT values


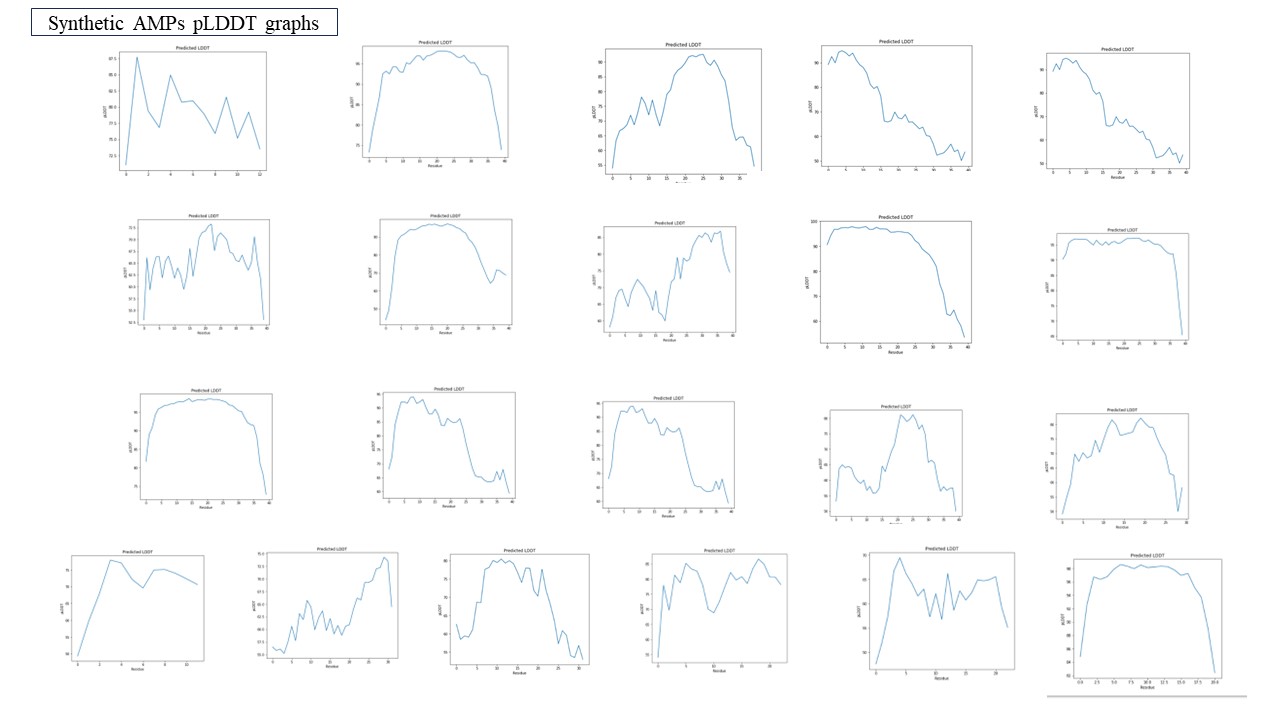

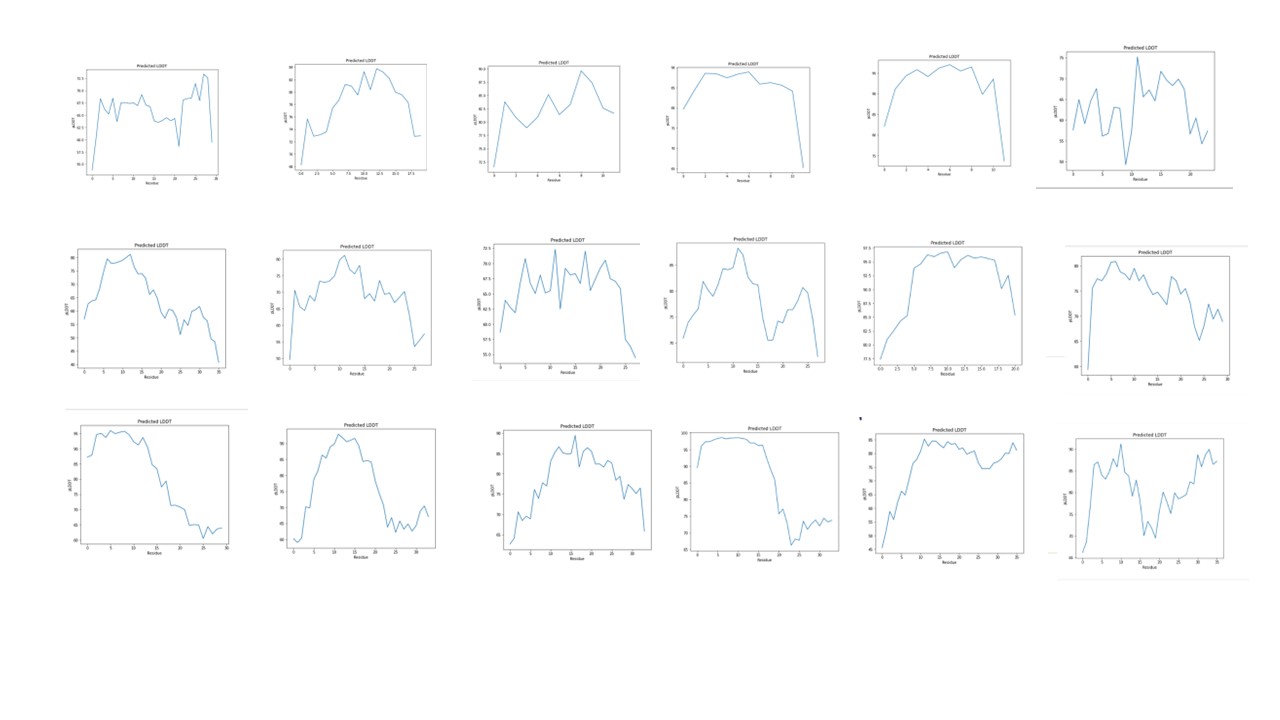

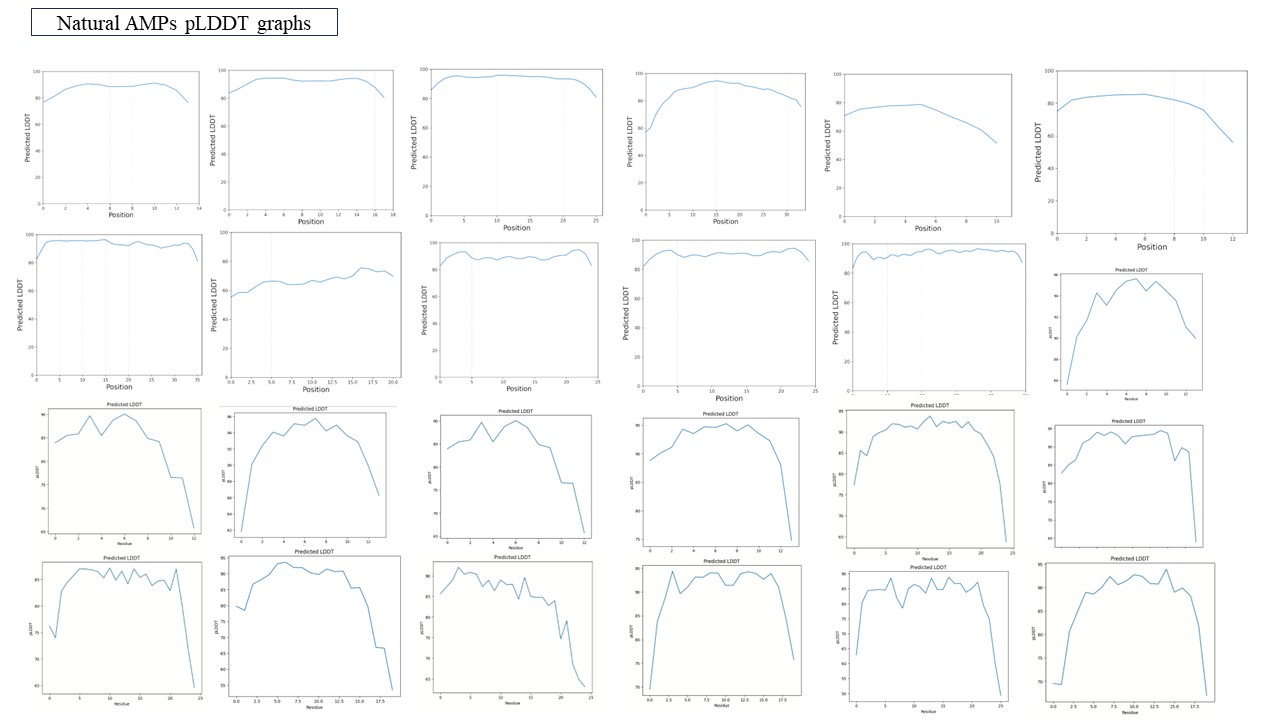

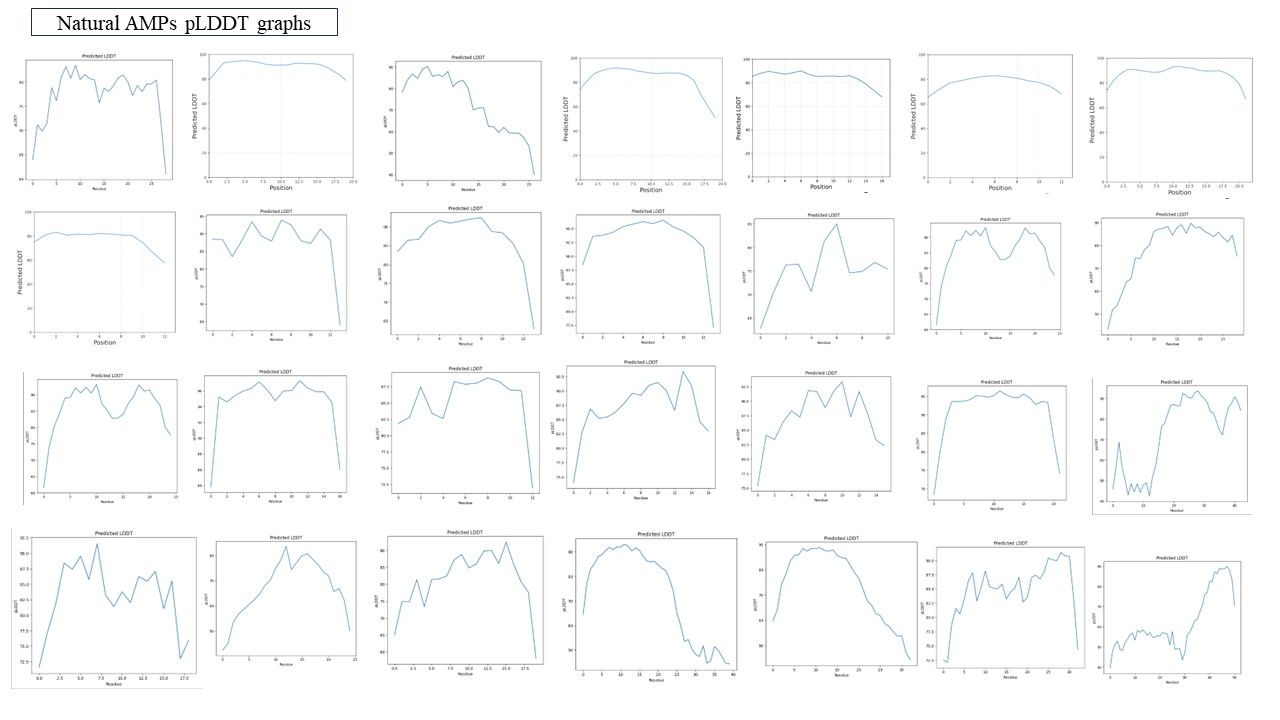

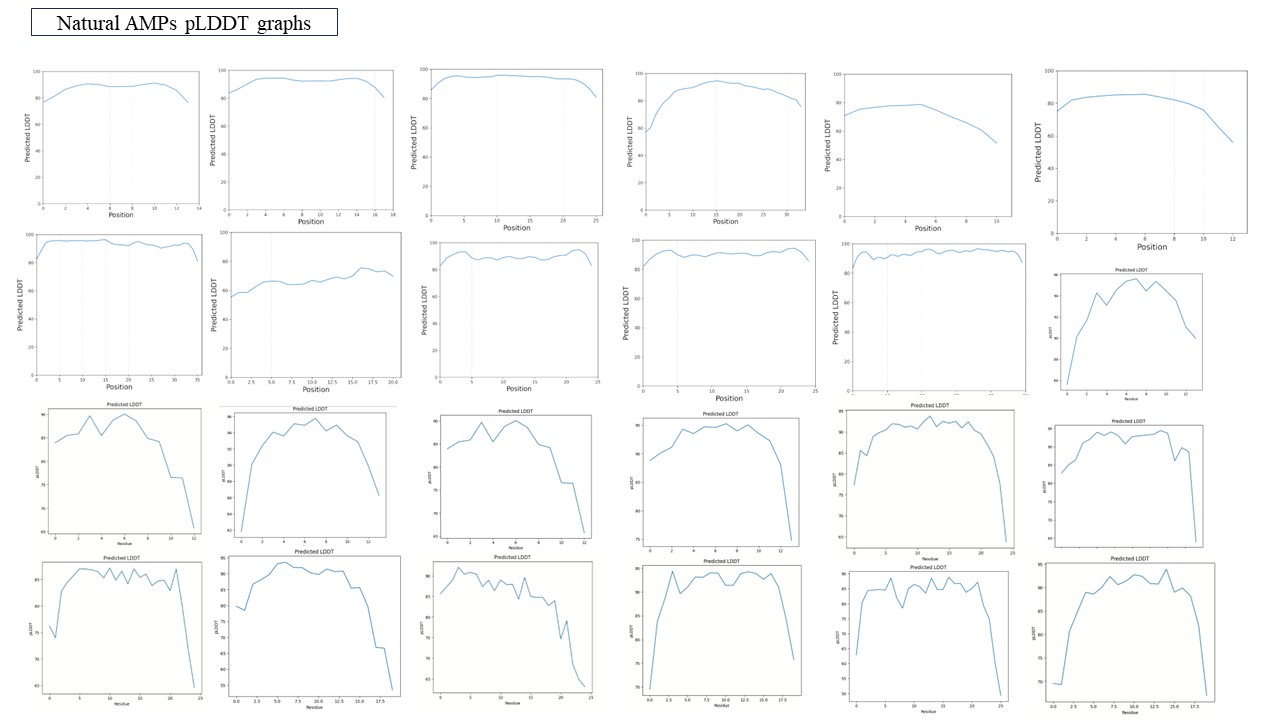

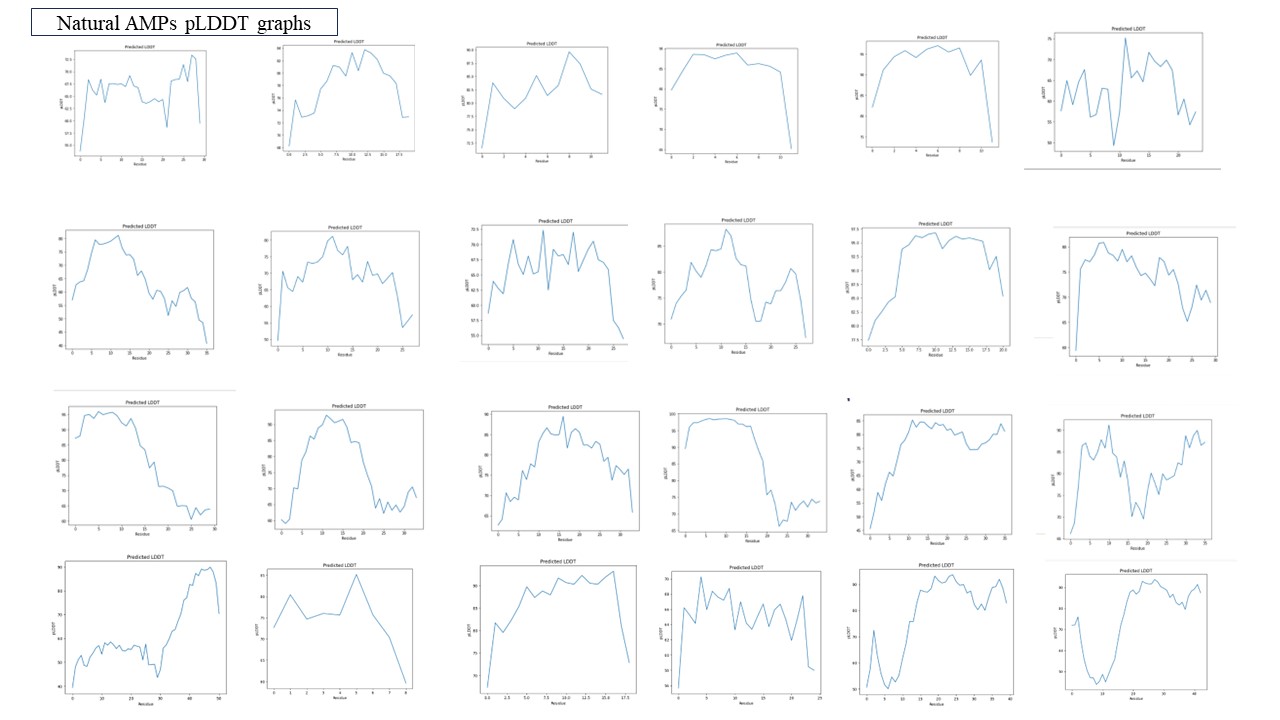

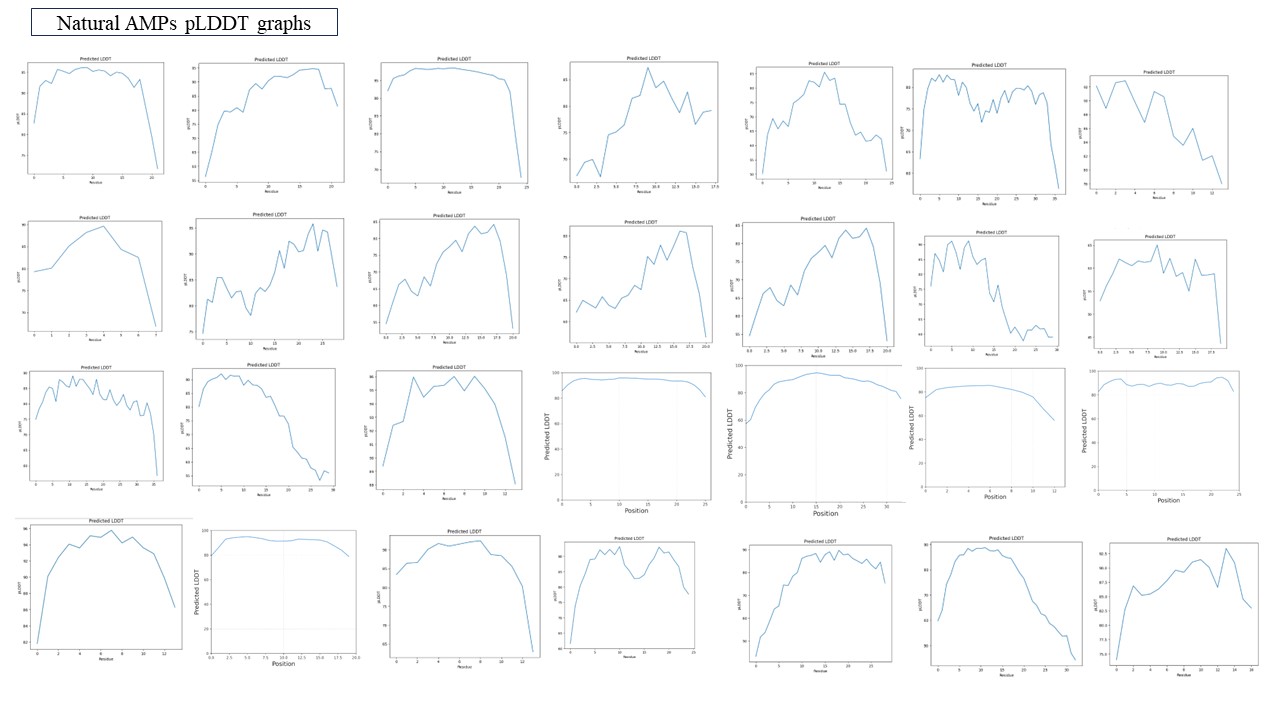

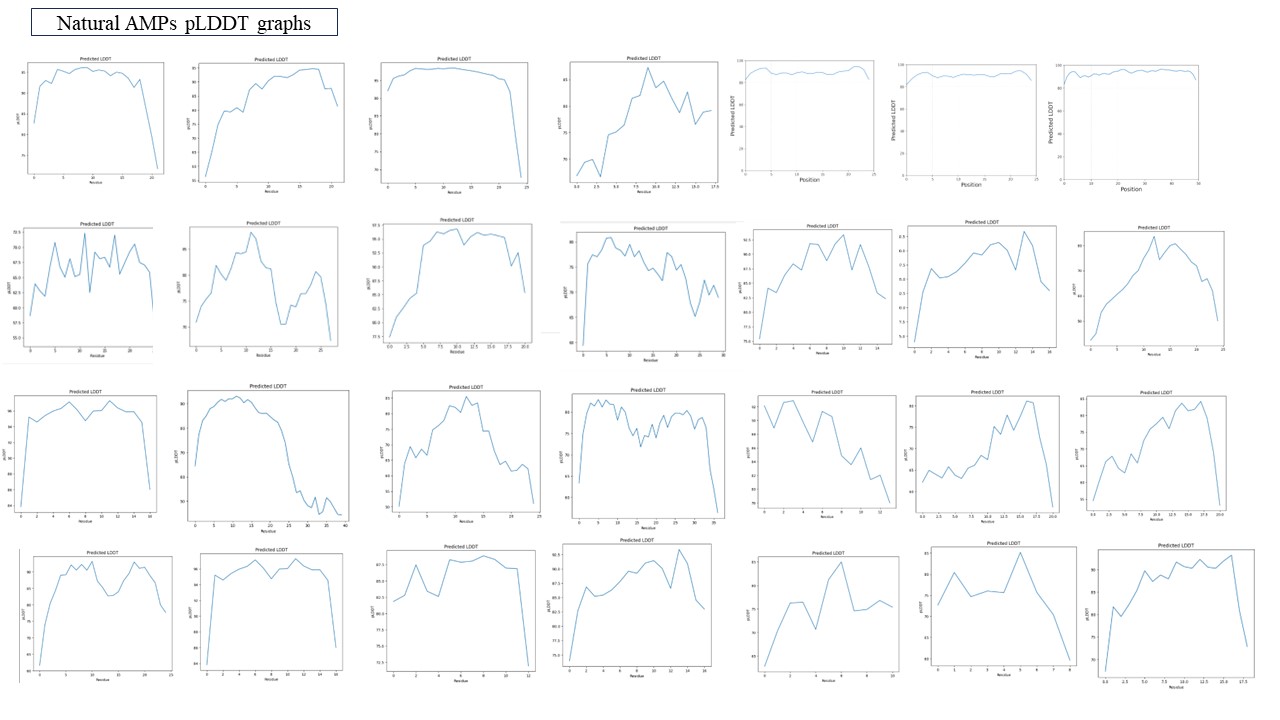

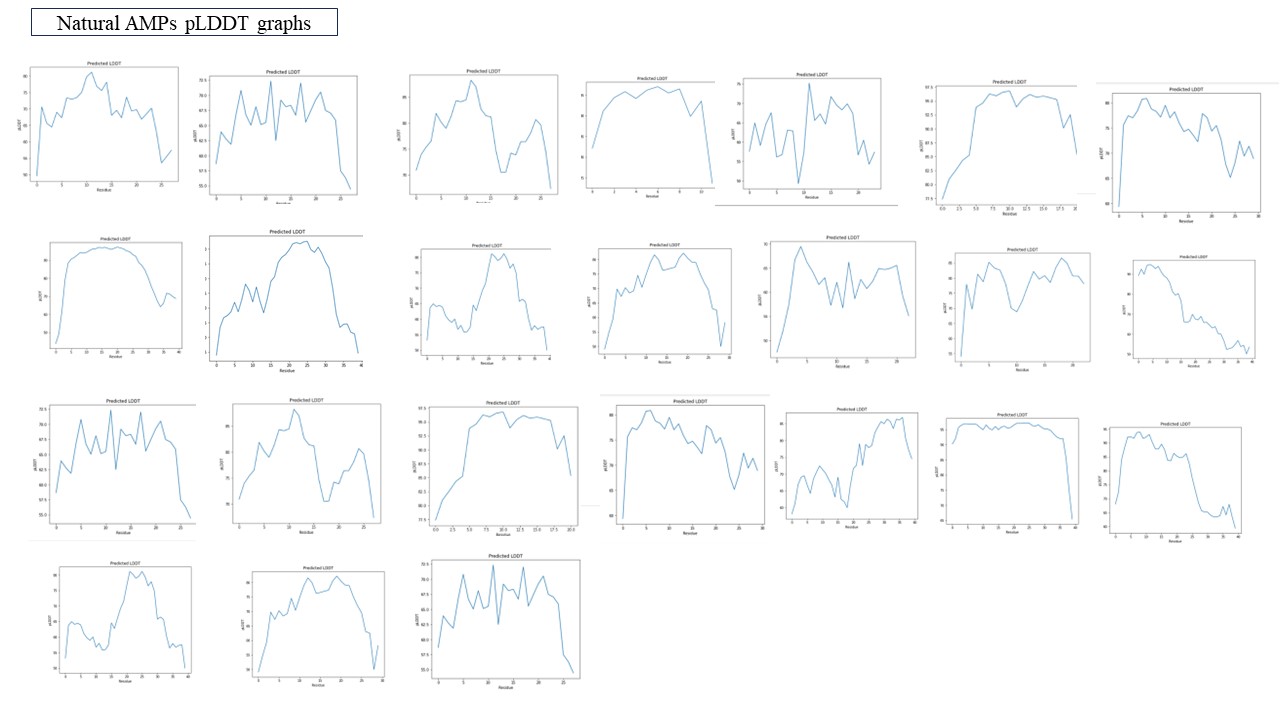

Supplement: baae096_Supp [file baae096_supp.zip › suppl_data/S3-Supporting Information File.docx]
